# Post-Cooking Quality Deterioration of Wheat (*Triticum durum*) During Bulgur Production

**DOI:** 10.3390/foods15172983

**Published:** 2026-08-25

**Authors:** Betul Bay-Yilmaz, Nuzhet Turker, Mustafa Bayram

**Affiliations:** 1 Department of Food Engineering, Faculty of Chemical and Metallurgical Engineering, Yildiz Technical University, Istanbul 34210, Türkiye; 2 Department of Food Engineering, Faculty of Engineering, Mersin University, Mersin 33343, Türkiye; nuzhet.turker@gmail.com; 3 Department of Food Engineering, Faculty of Engineering, Gaziantep University, Gaziantep 27310, Türkiye; profdrmusbay@gmail.com

**Keywords:** post-cooking holding, off-odor onset, lipid oxidation, microbial growth kinetics, relative odor activity value (ROAV), bulgur

## Abstract

This study characterized the pattern and timing of post-cooking quality deterioration in durum wheat held at 25, 35, and 45 °C by performing physicochemical, microbiological, and volatile compound analyses combined with a sensorially defined off-odor onset endpoint. The bulk moisture content remained high throughout holding at all temperatures. Titratable acidity increased from 1.49 to 2.15, and pH at off-odor onset decreased from 6.63 to 6.49 with increasing temperature. Off-odor was detected after 50, 45, and 34 h at 25, 35, and 45 °C, respectively. Microbial growth was fastest at 35 °C (μ_max_ = 0.0651 h^−1^). Thiobarbituric acid reactive substances (TBARS) values were slightly lower at 45 °C than at 25 °C, despite the shorter holding duration, whereas lipid oxidation-derived volatile aldehydes declined more sharply over the same comparison. This pattern, together with the earliest off-odor onset occurring at 45 °C, suggests that non-lipid-derived volatiles increasingly drive off-odor perception as holding temperature increases. Gas chromatography–mass spectrometry (GC–MS)-based relative odor activity value (ROAV) analysis identified 2-methoxy-4-vinylphenol, 2,4-decadienal, 2-nonenal, 2,3-butanedione, nonanal and 2-methoxyphenol as the principal contributors to the aroma profile. These findings indicate that off-odor development during post-cooking holding results from a temperature-dependent interplay between microbial and chemical deterioration pathways and provide a basis for optimizing holding time and temperature to delay off-odor onset and limit quality losses in bulgur production.

## 1. Introduction

Bulgur is a nutrient-rich whole-grain product made from durum wheat (*Triticum durum*). Bulgur production involves several processing steps: cleaning, cooking, drying, tempering, dehulling, milling, and classifying according to particle size [1,2]. Bulgur is increasingly recognized as a nutritious and health-promoting cereal-based food, owing to its high content of dietary fiber, resistant starch, B-group vitamins, minerals, and phenolic compounds [3]. Nonetheless, bulgur is highly susceptible to deterioration under the high-moisture conditions used during processing and post-cooking holding [4].

Quality deterioration during post-cooking holding is due to a complex interplay of physical, chemical, and microbiological factors, including moisture migration, lipid oxidation, aroma loss, and the proliferation of spoilage and pathogenic microorganisms [5]. In cereal-based systems, more broadly, starch retrogradation and moisture migration at both macroscopic and molecular levels have been described as primary physical mechanisms underlying textural and quality changes after cooking [6]. Research employing advanced analytical methods, such as low-field nuclear magnetic resonance, has demonstrated that water in cooked cereal systems is partitioned into bound, weakly bound, and free fractions. Additionally, extended storage weakens the interactions between water and the starch–protein matrix, increasing the proportion of free water [6,7]. Empirical models, such as the Peleg, modified Page, and logarithmic equations, together with Fickian diffusion approaches, have been used to describe, predict, and optimize moisture dynamics in cereal systems [1].

Cooked wheat is held in a surge tank between the cooking and drying operations of bulgur production. Free fatty acids rapidly degrade via lipase-mediated hydrolysis and autoxidative reactions under the high-moisture and -temperature conditions (>40%, >80 °C) of the post-cooking holding stage, generating volatile aldehydes and alcohols, such as hexanal, nonanal, and 1-octen-3-ol [8,9]. These oxidation products contribute to the development of bitter, stale, and undesirable off-odors [10,11]. Furthermore, storage under high-moisture and high-temperature conditions creates an environment that supports the proliferation of microorganisms, especially spore-forming bacteria such as *Bacillus cereus* and molds including *Aspergillus* spp. This, in turn, results in microbial spoilage, which leads to pH reduction and product rejection [4,5,12].

Moisture migration, lipid oxidation, and microbial spoilage have been investigated separately in rice- and wheat-based cereal systems; however, few studies have examined how these pathways interact under the high-moisture, high-temperature conditions characteristic of post-cooking holding in industrial bulgur production, or how their relative contributions shift with holding temperature and time. This study addresses this gap by conducting physicochemical, microbiological, and volatile compound analyses combined with a defined sensory endpoint: off-odor onset. A trained sensory panel sets this sensory endpoint, which is used to characterize the pattern and timing of quality deterioration in durum wheat during post-cooking holding.

Cooked durum wheat held at 25, 35, and 45 °C was analyzed to determine its bulk moisture content, physical properties, color, and titratable acidity. Furthermore, TBARS assays, GC–MS-based volatile and odor-activity profiling, and microbial growth kinetics analyses were performed until the onset of off-odor was sensorially determined at each temperature. Empirical kinetic models (sigmoid and Hill) were applied solely as descriptors of temporal trends. By associating the temporal patterns of lipid oxidation and microbial growth with the timing of off-odor onset, this study aims to clarify the relative contribution of chemical and microbiological pathways to deterioration at different holding temperatures, providing a basis for optimizing post-cooking holding conditions to delay off-odor onset and limit quality losses in bulgur production.

## 2. Materials and Methods

### 2.1. Wheat Cleaning and Cooking Procedures

*Triticum durum* wheat (Zivego) was provided by Simaş Bulgur Company (Gaziantep, Türkiye). The grains were stored in the dark at 5 °C until use.

Wheat (1000 g) was sieved to remove impurities, and lighter grains were separated using an aspirator. Distilled water was first boiled (97 °C, corresponding to the laboratory’s altitude), and 1000 g of wheat was then immersed in the boiling water. The temperature was monitored using a thermocouple and maintained at 97 ± 1 °C. The mixture was gently stirred every 5 min to ensure uniform distribution and prevent sticking. Cooking was continued for 60 min to achieve complete starch gelatinization, following the method described by Bayram [13].

### 2.2. Determination of Particle Density and Interstitial Void Fraction

Particle density and interstitial void fraction were determined for cleaned raw wheat and cooked wheat to characterize the packing structure of the grain mass and provide a quantitative basis for evaluating residual interstitial oxygen availability within the sealed containers used in the holding trials (Section 2.3). Particle density ($\rho_{p}$) was determined using a pycnometer with xylene as the immersion liquid (density, d = 0.85 g/cm^3^), according to the following equation [14]:

(1) $$\rho_{p}=\frac{\left(m_{s}-m_{0}\right)xd}{\left(m_{1}-m_{0}\right)-(m_{s_{1}}-m_{s})}\;$$

where m_s_ is the weight of the pycnometer containing the sample (g), m_0_ represents the weight of the empty pycnometer (g), m_1_ denotes the weight of the pycnometer filled with xylene (g), and $m_{s_{1}}$ stands for the weight of the pycnometer containing both the sample and xylene (g). Measurements were performed in triplicate.

Bulk density ($\rho_{p}$), expressed as hectoliter weight, was determined according to TS EN ISO 7971-3 [15]. The interstitial void fraction (ε) was calculated as:

(2) $$\in =(\rho_{p}-\rho_{b})/\rho_{p}\;$$

where $\rho_{p}$ denotes the particle density, and $\rho_{b}$ is the bulk density of the sample.

### 2.3. Assessment of Changes in Wheat Quality During Post-Cooking Holding

A single batch of cooked durum wheat was homogenized and divided into portions placed in sterile polypropylene sample containers (120 mL), each filled to the rim to minimize free headspace. Containers were assigned to a single holding temperature and time, and each container was opened only once, at its designated sampling time, so that every sample represented a closed holding system up to the point of analysis.

Samples were placed on the middle shelf and incubated in a natural convection laboratory incubator (EN 500, Nüve, Ankara, Türkiye). The three holding temperatures (25, 35, and 45 °C) were selected to represent a progressively increasing temperature scenario that may be encountered during industrial bulgur handling. These temperatures reflect conditions ranging from typical ambient storage to elevated temperatures arising from seasonal warming, residual process heat, or prolonged holding because of inadequate cooling.

The post-cooking holding step was designed as a controlled delayed-drying scenario, which is particularly relevant to industrial practice during hot summer periods, when elevated ambient temperatures, drying-line delays, equipment downtime, or temporary batch accumulation may prolong the residence time of high-moisture cooked wheat before drying. Therefore, the selected holding conditions allowed us to evaluate the deterioration risk and off-odor onset of cooked wheat under controlled temperature conditions.

Samples were collected at five holding time points (0, 10, 20, 30, and 50 h) at each temperature. The moisture content, pH, titratable acidity, color, TBARS, microbial growth, and volatile flavor compound analysis (GC–MS/VFCA) were analyzed at every time point to characterize the deterioration kinetics throughout the holding period. The onset of off-odor was defined as the time point at which odor deterioration became sensorially perceptible by orthonasal olfaction. This point was first established in preliminary trials, in which samples were monitored at intermediate time intervals and an approximate onset window was identified at each temperature (32–36 h at 45 °C, 42–48 h at 35 °C, and 50 h at 25 °C). Based on these results, a panel of eight assessors (four females and four males) subsequently monitored samples in the main experiment at closer intervals to pinpoint the precise onset time. Immediately after opening each sterile, screw-cap container, panelists rated off-odor intensity using a 100-point unstructured line scale (0 = no perceptible off-odor; 100 = extremely strong off-odor). After a 15 min equilibration period at room temperature, panelists also assigned a qualitative descriptor to each sample, selected from four categories: fresh, slight stale/musty, distinct off-odor, and strong, grain-associated off-odor. Samples receiving an off-odor intensity score of 60 or above were classified as “unacceptable.” This approach was designed to maximize temporal resolution near the critical onset transition. The precise onset time was consistently identified as 34 h at 45 °C, 45 h at 35 °C, and 50 h at 25 °C. An additional sample was collected at the off-odor onset time points at 45 and 35 °C, independent of the five fixed sampling points above, and titratable acidity, pH, TBARS, microbiological, and GC–MS/VFCA analyses were conducted to characterize the deterioration stage. Off-odor onset at 25 °C coincided with the final fixed sampling point (50 h), so no additional sample was required. All samples were stored at −18 °C until analysis, and all analyses were performed in triplicate, using independent replicate containers, which were homogenized separately. Photographs of the samples were acquired under fixed lighting conditions and a consistent camera distance and background, with no post-acquisition image editing performed, to allow for a qualitative comparison of visual appearance across holding conditions.

### 2.4. Physical and Chemical Analyses

At each designated sampling time point, sample containers (*n* = 3) assigned to that specific temperature–time combination were opened, and the entire contents of each container were homogenized separately. The homogenized samples were used for all subsequent physical and chemical analyses.

The moisture (% wet base, w.b.; Method 44-15.02) [16], protein (% dry base, d.b.; Method 46-10.01) [17], and ash content (% d.b., Method 08-01.01) [18] of the raw and cooked wheat samples held at different temperatures were determined using standard methods.

The color of raw and cooked wheat was assessed by determining the CIELAB values: L* (100 = white; 0 = black), a* (positive = red; negative = green), b* (positive = yellow; negative = blue), and the yellowness index (YI), using a HunterLab ColorFlex instrument (Model No. 45/0, Reston, VA, USA) under D65/C10 conditions. The pH was measured at 20 °C using a pH meter (Jenway, 3010, Bibby Scientific Ltd., Stone, Staffordshire, UK). Titratable acidity was determined following the procedure described in [19].

TBARS analysis of cooked wheat samples was performed according to the method described by Bozkurt [20]. The ground sample (2 g) was extracted three times with 10 mL of 0.4 M perchloric acid, and the combined extract was adjusted to a final volume of 25 mL. Following centrifugation at 1790× *g* for 5 min, 1 mL of the supernatant was combined with 5 mL of TBA reagent and heated in a boiling-water bath for 35 min. After cooling, absorbance was measured at 538 nm using a UV/Vis spectrophotometer (SP-300nano, Optima, Tokyo, Japan). TBARS values were determined using a standard curve generated with malondialdehyde (MDA) solutions. All chemicals were sourced from Sigma-Aldrich (Steinheim, Germany).

### 2.5. Mathematical Modeling of the Data

Three mathematical models were used to determine the temporal changes in total moisture content during post-cooking holding (Table 1) [13].

In these equations, y denotes the measured total moisture content (% w.b.), while x stands for the holding time (h). The parameters y_0_ and a share the same unit as moisture content (% w.b.). The parameter x_0_ indicates the time corresponding to the midpoint or inflection region of the fitted curve, and is expressed in hours (h). In the sigmoid model, b serves as a time-scale parameter, which governs the steepness, expressed in h. In the Hill model, b is a dimensionless empirical parameter that controls curve steepness, and c denotes the half-response time, expressed in h.

The time-dependent change in titratable acidity during post-cooking holding was described using an exponential model [5]:

(5) $$y=y_{0}+a\left(1-e^{-bt}\right)$$

where y_0_ is the initial titratable acidity at t = 0, a denotes the asymptotic change in acidity over the holding period, and b represents the rate constant governing the approach to the saturation value.

The growth kinetics of the total viable counts (TVCs) in wheat during post-cooking holding at 25, 35, and 45 °C were described using the modified Gompertz model [6]

(6) $$\log N_{t}=logN_{0}+a*exp\left[-exp(-B*\left(t-X_{c})\right)\right]$$

where N_t_ is the colony count at time t, N_0_ denotes the initial colony count at t = 0, a represents the theoretical difference between the initial microbial count and the model-predicted maximum population, B stands for the relative maximum growth rate at time X_c_ (h^−1^), X_c_ symbolizes the time required to reach the maximum growth rate, and t is the holding time (h). The maximum growth rate (μ_max_) and the duration of the lag phase (λ) were subsequently derived from the fitted parameters as:

(7) $${\;µ}_{max}=a*B/e\;$$

(8) $$\lambda =X_{C}-(\frac{1}{B})\;$$

where µ_max_ is the maximum growth rate (1/h), denoted as e = 2.7182, and λ represents the adaptation phase (h). Nonlinear least squares regression analyses were performed in SigmaPlot 11 to estimate equation parameters for all models.

### 2.6. Extraction of Volatile Compounds

Volatile compounds were extracted using a solid-phase microextraction (SPME) apparatus (Model 57330-U, Supelco, Bellefonte, PA, USA) with a divinylbenzene-carboxen-polydimethylsiloxane (gray) fiber (50/30 μm thick, Model 57328-U, Supelco) as the absorbent. This is considered the standard method for extracting volatile compounds from cereals [21,22]. For extraction, 6 g of ground bulgur was placed in a 30 mL vial sealed with a silicone septum and heated in a water bath. The fiber absorbed volatiles from the headspace at 70 °C for 120 min. The SPME fiber was thermally desorbed at 250 °C for 5 min in the GC–MS injection port before reuse [22].

### 2.7. GC–MS Analysis

GC–MS analysis was performed using a Perkin Elmer Clarus 500 instrument (Perkin Elmer, Shelton, CT, USA). Volatile compounds were separated on a SUPELCOWAX 10 capillary column (30 m length, 0.25 mm internal diameter, 0.25 μm film thickness; N316551, Perkin Elmer). Helium was used as the carrier gas at a flow rate of 1.5 mL/min. The oven temperature program was as follows: 40 °C for 4 min, increased to 90 °C at 3 °C/min, then to 130 °C at 4 °C/min and held for 4 min, and finally increased to 240 °C at 5 °C/min and held for 8 min [22]. The injection port was operated in splitless mode at 250 °C. The MS electron energy was set to 70 eV in EI+ mode, with a source temperature of 180 °C and a mass range of 30–350 °C. The Wiley and NIST/EPA/NIT libraries were used for peak identification. Volatile compound concentrations were expressed as percentages. All analyses were conducted in triplicate.

### 2.8. Assessment of Relative Odor Activity Values

Odor thresholds (OTs) for the identified volatile compounds were obtained from van Gemert’s [23] compilation. This compilation aggregates threshold data reported across multiple independent studies for each compound. Odor thresholds determined in air (mg/m^3^) were prioritized. If the air-phase threshold was unavailable for a given compound, the corresponding water-phase threshold was used. For compounds with multiple reported thresholds, a representative value was selected based on the most consistent and reliable reported data. Individual volatile compounds were then evaluated for their contribution to the overall aroma profile, quantified using the ROAV approach [24], which evaluates the perceptual significance of volatiles by integrating concentration data with olfactory thresholds and is defined as,

(9) $${ROAV}_{i}=\left(\frac{C_{\%i}/T_{i}}{C_{\%max}/T_{max}}\right)\times 100$$

where C_%i_ denotes the relative concentration of compound i, expressed as peak area percentage through normalization; T_i_ is the odor threshold of compound i (mg/m^3^, air); C_%max_ represents the highest relative concentration among all volatiles; and T_max_ stands for the odor threshold of the dominant compound. Odorants with ROAV ≥ 1.0 are key aroma compounds (i.e., they have a decisive impact on overall odor). Conversely, those with 0.1 ≤ ROAV < 1.0 are modifier compounds that enhance aromatic nuances.

### 2.9. Statistical Analysis

Statistical analysis was performed using one-way analysis of variance (ANOVA) at a significance level of α ≤ 0.05. Multiple range (Duncan) tests were performed using SPSS Statistics v.22 (IBM Co., Chicago, IL, USA) to determine significant differences. Aroma profile clustering was visualized through heat map analysis using Phython 3.12.13 in Google Colab (CA, USA).

## 3. Results and Discussion

### 3.1. Effects of Cooking on the Physicochemical Properties of Triticum durum

Cooking is a critical stage in which the physical and chemical properties of wheat kernels undergo substantial changes. Its primary objective is to achieve starch gelatinization without deforming the wheat kernel; thus, the optimal cooking time should be determined. A comparison of raw *Triticum durum* with cooked samples revealed significant differences (*p* ≤ 0.05) in moisture content (Table 2). The marked increase in moisture, from 6.85% in the raw grain to more than 50.17% in the cooked matrix, can be attributed to the extensive hydration needed for starch gelatinization. By contrast, the decrease in the ash content is likely due to the leaching of water-soluble minerals into the boiling water. On the other hand, the marked shifts in the L*, a*, b*, and YI values reflect the characteristic darkening and amber-yellow color of bulgur.

Particle density and the interstitial void fraction were also determined for cleaned raw and cooked wheat to characterize the packing structure of the grain mass and provide a quantitative basis for evaluating residual interstitial oxygen availability within the sealed containers used in the experiments. Cleaned raw wheat exhibited a bulk density of 83.93 ± 0.06 kg/hL, a particle density of 1318.80 ± 2.56 kg/m^3^, and a void fraction of 0.3912 ± 0.0026. By contrast, cooked wheat showed a bulk density of 72.65 ± 0.14 kg/hL, a particle density of 1202.16 ± 3.77 kg/m^3^, and a void fraction of 0.4126 ± 0.0133. The decrease in bulk and particle density after cooking is consistent with the hydration-induced swelling and structural loosening of the grain during starch gelatinization. By contrast, the increase in the void fraction reflects the change in the packing geometry of the cooked kernels. The abovementioned values indicate that approximately 39–41% of the packed sample volume consisted of interstitial air, confirming that the sealed containers used in the experiments retained substantial residual oxygen. This physical characterization provides a quantitative basis for interpreting the aerobic/microaerophilic nature of the deterioration processes.

The one-way ANOVA results for cooked durum wheat samples revealed significant changes (*p* ≤ 0.05) in moisture content and color parameters (L, a, b*, and YI) during the post-cooking holding period. By contrast, the protein content was not significantly affected by cooking (*p* > 0.05). The pH decreased during post-cooking holding, and Duncan’s multiple comparison analysis revealed a separation between the pHs of the initial samples and those kept at different temperatures after cooking, indicating that holding temperature and duration significantly (*p* ≤ 0.05) affected pH evolution.

GC–MS analysis of cooked *Triticum durum* revealed a volatile fraction dominated by medium- and long-chain hydrocarbons, with dodecane, tetradecane, heneicosane, and eicosane exhibiting the highest relative abundances as detailed in Section 3.7. This observation aligns with previous findings. For instance, Yousif et al. [22] reported that hydrocarbons, particularly dodecane, produced through complex thermal lipid degradation pathways are major contributors to the characteristic flavor of bulgur.

### 3.2. Evaluation of Moisture Content During Post-Cooking Holding

The moisture content of wheat increases to approximately 40–50% after cooking. This elevated moisture content favors microbial growth and affects subsequent processing steps in bulgur production. Thus, products stored in silos after cooking, especially during warmer periods, may experience deterioration in color, flavor, and odor.

Moisture content is a primary factor influencing the structural properties of bulgur. Inadequate starch gelatinization during cooking results in rapid texture deterioration during preparation and serving [25]. This deterioration mainly occurs during the post-cooking holding period and causes both quality and economic losses. Here, the moisture content was determined gravimetrically and expressed as bulk moisture content (% w.b.). The bulk moisture content remained high and stable throughout post-cooking holding (52.5–54% w.b.). Accordingly, the analysis reflects temporal changes in measured moisture content rather than spatial moisture distribution, intragranular diffusion, or actual moisture transport within individual kernels.

Although Fickian diffusion-based models can provide mechanistic insights into hydration and rehydration processes, their reliable application requires assumptions such as monotonic moisture change, well-defined geometry, constant effective diffusivity, and a clearly defined equilibrium moisture content. In the present study, bulk moisture was already high and stable at the onset of holding, so no sustained concentration gradient was available to drive Fickian diffusion. The limited and partly non-monotonic changes in bulk moisture content, together with the heterogeneous structure of cooked durum wheat kernels, did not allow a reliable estimation of effective diffusivity. Moreover, under the high-moisture post-cooking holding conditions, capillary redistribution, swelling, and structural relaxation may contribute to the observed moisture behavior [26], further limiting the applicability of a simplified Fickian approach. Therefore, Fickian diffusion modeling was not used to infer physical transport coefficients.

Thus, sigmoid and Hill models were evaluated as empirical descriptors of the temporal changes in total moisture content within the experimental holding period. According to Peleg [27], such empirical hydration and moisture-sorption models are descriptive and are not derived from physical mass-transfer principles; hence, their parameters are model-dependent and should not be interpreted as diffusion coefficients, mass-transfer parameters, or indicators of moisture transport within the grain. Because the bulk moisture content changed only slightly over the holding period, the fitted shape and inflection parameters were sensitive to the limited number of data points and the narrow response range and were treated as descriptors of apparent curve shape. The corresponding fitting results are provided in the Supplementary Material (Figure S1 and Table S1). The observed deterioration proceeded under sustained high moisture. As noted by [27], the physical meaning of a true equilibrium moisture condition in high-moisture cooked cereal systems undergoing biochemical and/or microbial changes may be unclear, supporting our decision to report the measured moisture content directly rather than through equilibrium-based transport models. Although bulk moisture content reflects the overall amount of water under the closed batch-holding conditions used here, water activity (aw) is more directly related to microbial growth potential and would provide a more precise assessment of microbial stability. However, water activity values and spatial moisture profiles were not obtained. Therefore, future studies should incorporate water activity measurements alongside spatially resolved techniques, such as low-field nuclear magnetic resonance (LF–NMR) or structural analyses, to better evaluate water availability and moisture distribution within cooked wheat kernels.

### 3.3. pH and Titratable Acidity Changes in Wheat During Post-Cooking Holding

The pH values during the post-cooking holding stage ranged from 7.09 to 6.29. The pHs measured at holding temperatures of 25, 35, and 45 °C at the initial detection of off-odor were 6.63, 6.54, and 6.49, respectively. Following the onset of off-odor, the pH of the medium gradually decreased with increasing temperature and time. The observed decrease in pH aligns with previous findings in cereal-based systems, where microbial growth leads to organic acid production and subsequent acidification [28]. Similarly, the pH decreased slightly from 6.42 to 6.35 during the first 18 h for fresh brown rice noodles held at 37 °C, followed by a more pronounced acidification phase. By contrast, samples kept at 25 °C showed a rapid pH drop to 5.99, demonstrating that holding temperature critically influences acidification kinetics in cereal-based products [7]. Additionally, recent studies have identified a strong correlation between decreasing pH values and increasing total plate counts in bulgur and other cereal-based products [4,29].

**Figure 1:**
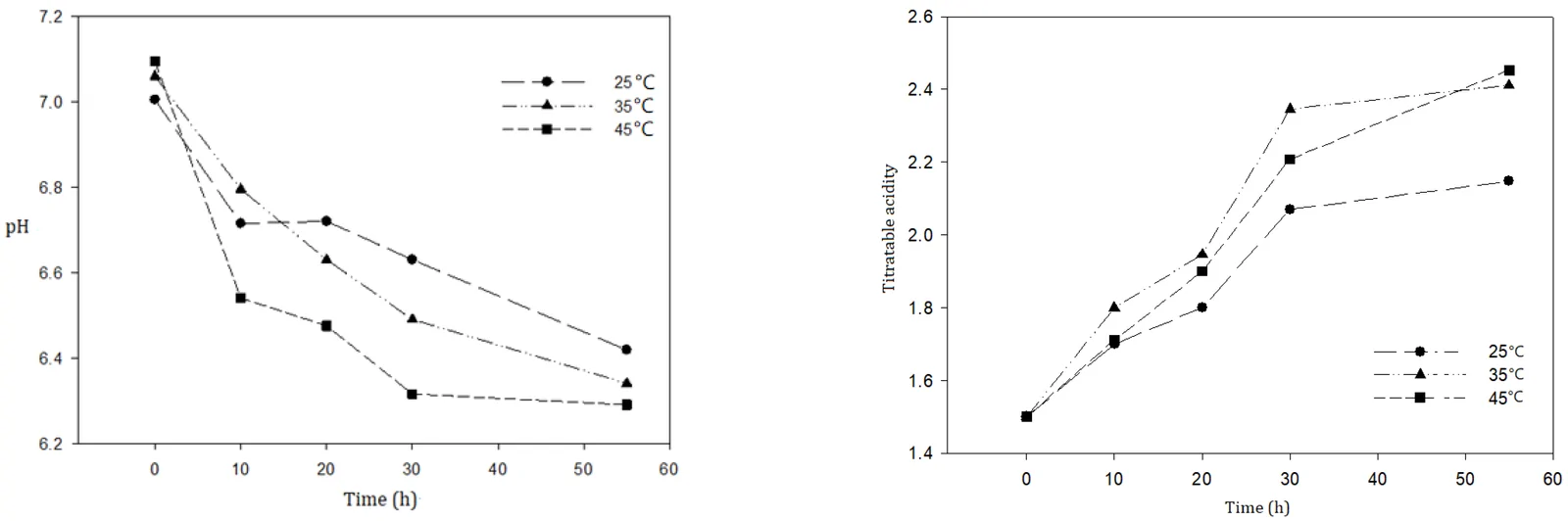
pH and titratable acidity profiles of wheat samples during post-cooking holding at 25, 35, and 45 °C.

Here, titratable acidity increased concurrently with the decrease in pH (Figure 1). This trend reflects intensified microbial activity and elevated acid production. Titratable acidity rose from approximately 1.49 at the start of the holding period (t = 0) to 2.15 after 50 h. This observed decrease in pH and simultaneous increase in titratable acidity can be primarily attributed to intensive microbial activity and carbohydrate metabolism within the product matrix [28,30]. This mechanism has been widely reported in cereal-based systems, particularly in fresh noodle matrices, and is likely applicable to cooked wheat samples. The inverse relationship between pH and titratable acidity is consistent with the increased microbial load observed in the system, indicating that acidification is primarily driven by microbial metabolism during the post-cooking holding period. Therefore, the physicochemical and odor-related changes in the cooked wheat samples, particularly acidification and off-odor development, are likely driven by microbes [28,30].

**Figure 2:**
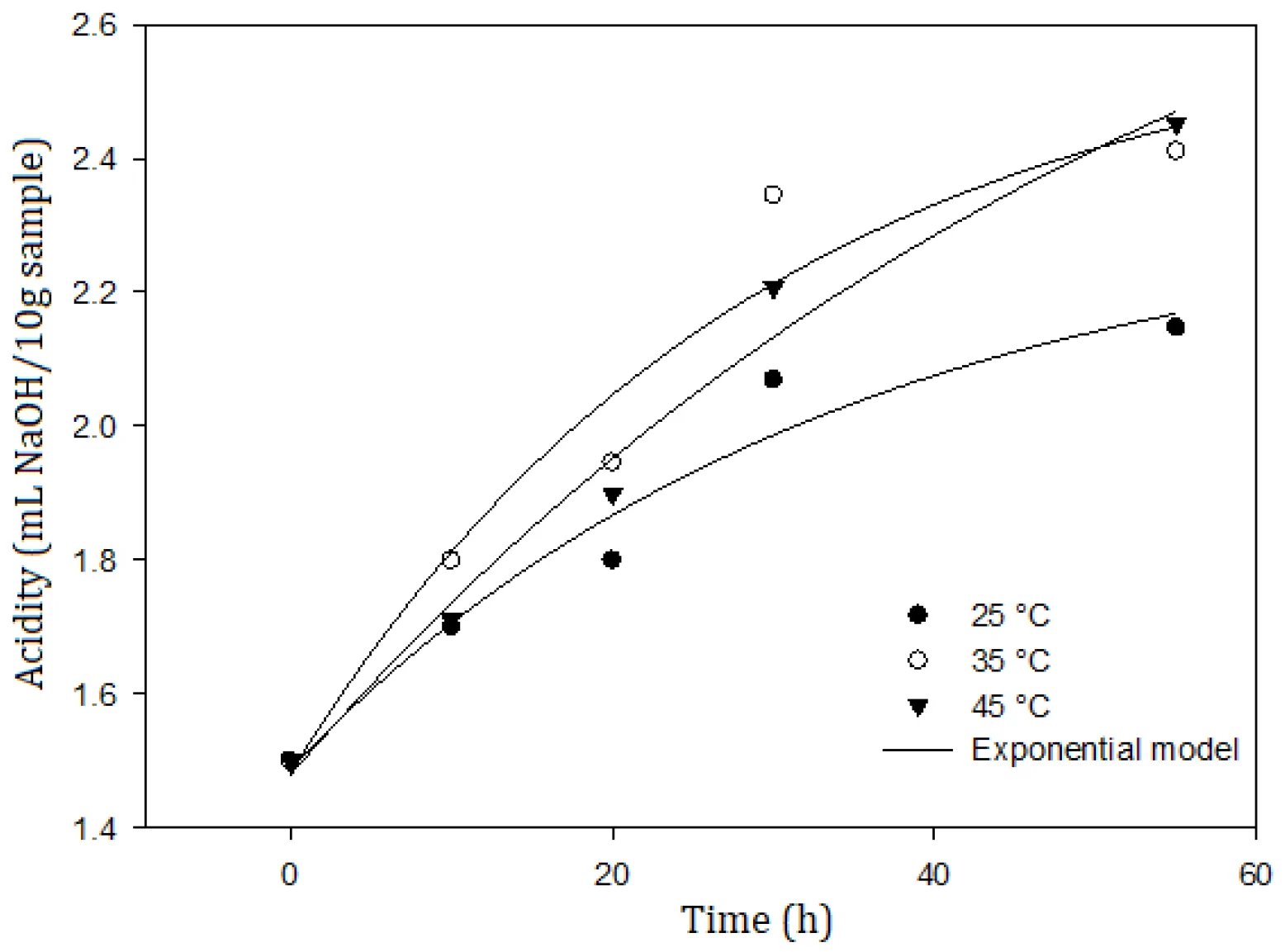
Exponential model fitting of titratable acidity as a function of post-cooking holding time for wheat samples kept at 25, 35, and 45 °C.

The time-dependent changes in the titratable acidity of the samples held at 25, 35, and 45 °C after cooking were analyzed using an exponential model (Figure 2). Table 3 presents the estimated model parameters. The goodness of fit of the model was evaluated. As a result, the high coefficients of determination (R^2^ > 0.949), coupled with remarkably low RMSE (<0.03) values, indicate that the exponential model adequately describes the acidification dynamics within the cooked wheat matrix.

In the exponential model, y_0_ denotes the initial acidity at t = 0, a represents the extent of acidity increase, and b reflects the rate at which the system approaches the asymptotic value. As discussed in our previous study [4], the exponential model is subject to several simplifying assumptions. In particular, acidification is assumed to proceed in a monotonic, unidirectional manner, a constraint that does not accommodate transient fluctuations or reversals potentially arising from microbial activity, enzymatic action, or environmental feedback within the food matrix. The model also presumes asymptotic convergence toward a saturation value, a pattern typical of concentration-driven or diffusion-limited kinetics and consistent with the nonchaotic behavior reported in other food systems.

Although this approach effectively captures the overall temporal trend, its nature remains descriptive because the fitted parameters (y_0_, a, and b) are treated as time-invariant and do not explicitly account for concurrent variations in moisture content, temperature, or microbial activity. These simplifications may constrain the model’s ability to fully represent the dynamics of complex, real food systems undergoing spoilage. Hence, model predictions should be validated against independent experimental data and, where appropriate, incorporate complementary mechanistic approaches.

The model parameters provided further insights into both the initial state of the system and the extent of deterioration at different temperatures. As shown in Table 3, y_0_ varied only slightly among temperature groups (1.479–1.488), confirming that the samples started the holding period with comparable acidity immediately after cooking. This observation suggests that the subsequent divergence in acidity profiles was primarily temperature-driven.

By contrast, clear temperature-dependent differences were observed for parameters a and b. The rate constant b, which reflects the kinetics of acid production, increased markedly from 0.298 at 25 °C to 0.739 at 45 °C, corresponding to more than a 2.5-fold increase in the acidification rate (Table 3). Similarly, a, which is associated with the extent of acid accumulation, increased from 0.844 to 1.610 with rising temperature. These changes indicate that elevated temperatures intensified the rate and overall magnitude of acid development within the cooked wheat matrix.

This exponential response can be interpreted in relation to the enhanced microbial and biochemical activity occurring at higher holding temperatures. Spoilage-associated microorganisms proliferate rapidly under such conditions, leading to the accelerated metabolism of available substrates and promoting the accumulation of acidic metabolites. Therefore, the substantially higher a* and b* values observed at 45 °C suggest that samples held at elevated temperatures underwent more intense and rapid deterioration.

Overall, the modeling results demonstrate that temperature plays a decisive role in shaping the kinetics of post-cooking acidification in bulgur. Higher incubation temperatures promoted a faster and more extensive increase in acidity, indicating reduced product stability during post-cooking holding. From an industrial perspective, these findings highlight the critical importance of rapid post-cooking cooling and drying because the simultaneous reduction in both moisture content (to below 10%) and product temperature is essential for enhancing product stability, extending shelf life, and preserving sensory quality [3].

### 3.4. Microbial Growth Kinetics of Cooked Wheat

The analysis of TVC data demonstrated that there were no visible colonies immediately after cooking (0 h), and initial microbial loads remained below 2 log CFU/g across all temperature groups. Raw cereal matrices are typically associated with diverse field-derived microorganisms; thus, the negligible microbial counts observed at 0 h suggest that cooking inactivated the vegetative microbial population present before processing [12].

Off-odor development did not align with the time corresponding to microbiological spoilage. Although the Gompertz model indicated that microbial populations exceeded the critical safety threshold of 10^6^ CFU/g within the initial 10 h of incubation, spoilage odors were detected substantially later: at 50 h at 25 °C, at 45 h at 35 °C, and at 34 h at 45 °C. However, a sharp increase in TVCs was observed after only 10 h of storage at 25, 35, and 45 °C, with populations rapidly rising to 6.31 ± 0.05, 6.58 ± 0.05, and 6.36 ± 0.0 log CFU/g, respectively. These findings are consistent with previous reports showing that the storage of high-moisture cooked cereal matrices under temperature-abused conditions not only enables rapid microbial proliferation but may also favor the germination of heat-resistant spores, particularly those of *Bacillus* spp., and fungal spores that survive the initial cooking treatment [12,31].

The microbial load of cooked foods should remain within recommended microbiological limits. Values at or above 10^6^ CFU/g generally indicate the onset of microbiological spoilage [28], beyond which the product is regarded as highly contaminated and unsuitable for consumption. The rapid and pronounced microbial proliferation observed here suggests that improper post-cooking holding temperatures rapidly compromise the microbiological stability of bulgur.

Table 4 presents the Gompertz model parameters used to examine the growth kinetics of TVCs in high-moisture wheat samples during post-cooking holding at 25, 35, and 45 °C at the off-odor onset. The model showed excellent agreement with the experimental data (R^2^ = 0.993–0.999). In addition, TVCs differed significantly (*p* ≤ 0.05) among the incubation temperatures.

The analysis of the model parameters revealed that the highest estimated specific growth rate (μ_max_) was 0.0651 h^−1^ at 35 °C. Previous research has demonstrated that although intensive cooking of cereal matrices generally inactivates vegetative cells, heat-resistant microbial spores, particularly those of *Bacillus* spp., may persist within the matrix [5]. Holding at 30–35 °C provides highly favorable conditions for the recovery and rapid logarithmic growth of surviving mesophilic flora, including potentially opportunistic spoilage-associated microorganisms [4]. The exceptionally high μ_max_ value observed at 35 °C implies a substantial accumulation of fermentation- and microbial degradation-related volatile markers, which were identified in the GC–MS profile. These included acetoin, 2,3-butanediol, hexanal, and 2-methoxy-4-vinylphenol. These results indicate that accelerated microbial growth at 35 °C likely contributed to the formation of these compounds.

By contrast, increasing the incubation temperature to 45 °C resulted in a drop in the specific growth rate (0.0352 h^−1^), indicating that the thermal tolerance of the mesophilic flora was exceeded and that elevated heat stress suppressed microbial metabolism. Although the highest μ_max_ was observed at 35 °C, the earliest onset of off-odor occurred at 45 °C. This discrepancy raises the possibility that, at 45 °C, nonenzymatic deterioration pathways, such as lipid autoxidation and the thermal degradation of phenolic acids, contributed proportionally more to off-odor onset than microbial metabolism [32]. This interpretation is supported by the volatile and physicochemical profile. The increase in hexadecanoic acid and high relative area of phenolic volatiles (2-methoxyphenol and 2-methoxy-4-vinylphenol) at 45 °C are both consistent with advanced oxidative and thermal degradation processes (Table 7). Therefore, we interpret the earlier off-odor onset at 45 °C as reflecting a shift in the relative balance between microbial and chemical deterioration pathways.

The adaptation (lag) phase (λ) was completed within a short time (0.234–0.245 h) across all post-cooking temperatures. This short lag phase is likely attributable to physicochemical changes in the wheat matrix. Thermal processing promotes starch gelatinization and weakens the protein matrix. By contrast, tempering induces the formation of cracks, pores, and capillary channels within the grain structure, enhancing mass transfer and reducing diffusion resistance [1]. These structural modifications increase the accessibility of water and soluble components within the matrix. The resulting porous structure provides an environment that is accessible to surviving heat-resistant microorganisms, facilitating their transition from the lag phase to the exponential growth phase. These observations agree with previous studies that showed that high-moisture cereal-based systems are highly susceptible to rapid microbial proliferation under temperature-abused conditions. For instance, TVCs in fresh noodle matrices have been reported to exceed 10^6^ CFU/g within 24 h at temperatures ≥ 25 °C, highlighting the susceptibility of such cereal-based systems to microbial deterioration [29].

In conclusion, the extremely short adaptation times together with the aggressive specific growth rate, observed particularly at 35 °C, demonstrate the susceptibility of the high-moisture cooked wheat matrix to microbial spoilage and degradation during the post-cooking holding period. These kinetic findings imply that the strict control of temperature and moisture during this stage is critical for maintaining microbiological stability and preventing the formation of enzyme- and microbe-derived off-odor compounds.

### 3.5. Color Changes During Post-Cooking Holding

The color changes observed in cereal-based products during processing and storage reflect complex physicochemical and biochemical reactions that influence product quality and consumer acceptance [5]. Here, such changes were evaluated in cooked wheat samples held at different temperatures. Dynamic changes in the color parameters of the samples are governed by diverse mechanisms that are activated depending on processing conditions and storage temperatures. These mechanisms include enzymatic and nonenzymatic pathways, such as oxidative reactions, Maillard-type processes, and structural modifications within the matrix [5,33].

The time-dependent changes in the color profiles of cooked wheat samples held at 25, 35, and 45 °C revealed the structural and biochemical mechanisms that occurred within the product matrix (Table 5). The lightness (L*) values increased significantly (*p* ≤ 0.05) at 25 °C, but no significant changes were observed at 35 or 45 °C (*p* > 0.05). For instance, the initial L* value of samples kept at 25 °C increased from 46.62 to 48.55 by the end of the post-cooking holding period. This increase in L*, reflecting whitening or opacity, has been reported in cooked cereal systems and is widely recognized as an indicator of starch retrogradation during storage [34]. The rearrangement and recrystallization of gelatinized starch components, particularly amylose and amylopectin, into more ordered structures [5,35] during post-cooking holding may have resulted in a more opaque and whitish appearance by reducing light transmission through the matrix.

**Figure 3:**
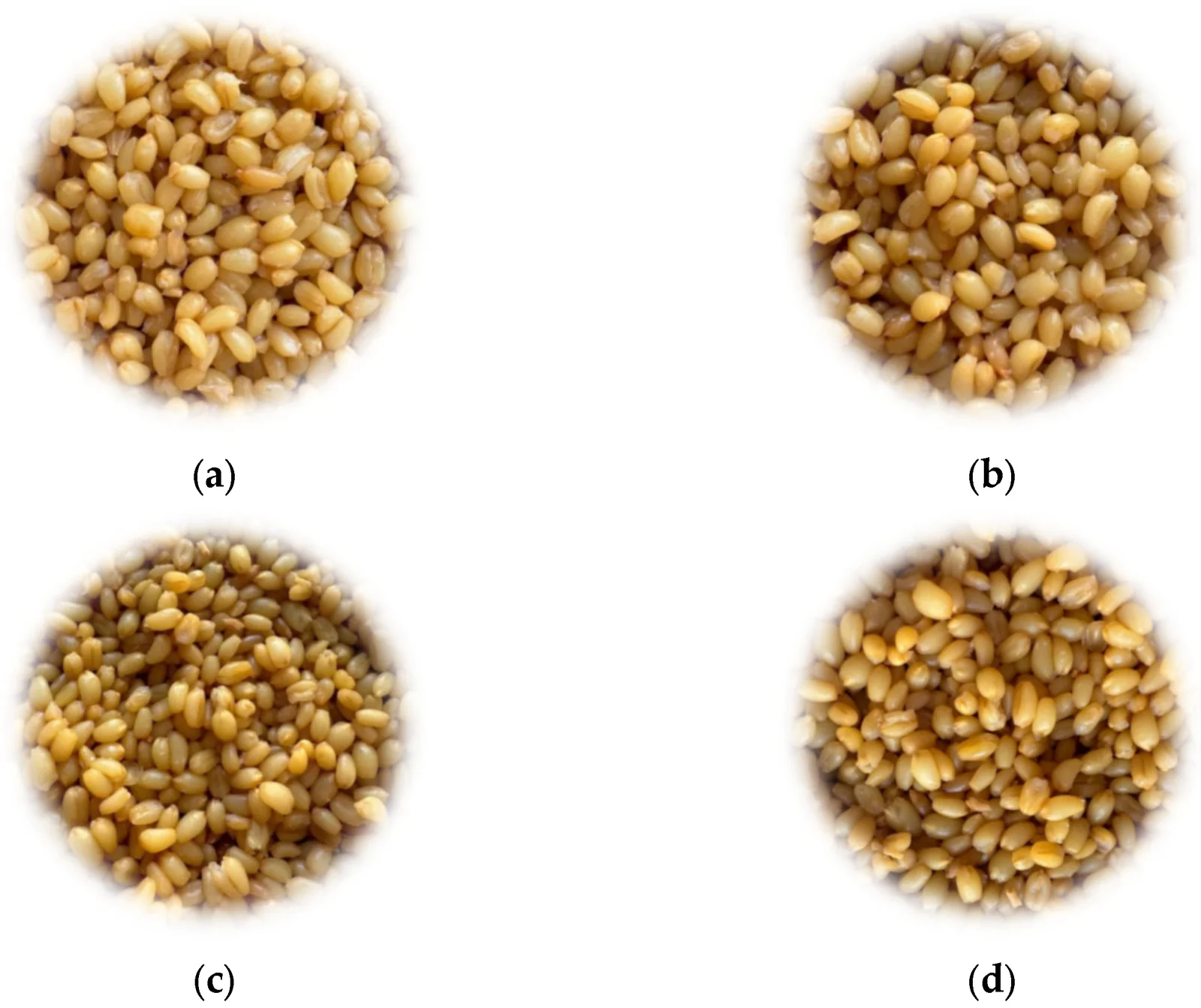
Cooked durum wheat samples at (**a**) t = 0 h and at off-odor onset following holding at (**b**) 25 °C, (**c**) 35 °C, and (**d**) 45 °C after cooking.

In contrast to the increase in L*, both a* and yellowness index (YI) declined throughout post-cooking holding. The initial a* values (7.76–7.95 at t = 0) decreased significantly to 7.55–7.49 across all temperatures by the end of the holding period (50 h). Previous studies have reported a decrease in a* values after cooking in bulgur-based products, indicating a reduction in redness as a result of thermal processing [36]. YI values, which initially ranged from 88.86 to 89.60, dropped to approximately 85.93–87.48 by the end of the holding period (Figure 3). The observed decrease in YI values can be attributed to the degradation of natural pigments, particularly carotenoids (e.g., lutein), which are responsible for the characteristic yellow color of bulgur and are highly susceptible to oxidative degradation during storage [37]. Previous studies have indicated that lipid oxidation produces reactive carbonyl compounds, which may accelerate the degradation of endogenous pigments and modify chromophoric structures in the product matrix, deteriorating the color [38].

At 25 and 35 °C, b* values remained fairly stable (*p* > 0.05), exhibiting only minor fluctuations or a slight decrease throughout storage. By contrast, samples kept at 45 °C showed a significant increase (*p* ≤ 0.05) in b* values, which rose from 30.30 to 32.70. This increase is likely due to temperature-induced nonenzymatic browning, particularly Maillard-type reactions, in which reducing sugars and free amino compounds produce yellow-brown reaction products [34]. Given the simultaneous decreases in a* and YI, the increase in b* at 45 °C may be explained by the combined effects of browning-related pigment formation, structural changes in the matrix, and modified light-scattering properties. Pekkirişci et al. [39] reported that b* values in firik bulgur usually increase after cooking. By contrast, regular bulgur types may exhibit different responses depending on their composition and processing conditions.

The color data demonstrate that post-cooking holding involved physical and chemical transformations within the product matrix. Decreases in a* and YI values across all groups may indicate oxidative pigment degradation. By contrast, the increase in L* at 25 °C implies enhanced opacity that may be consistent with starch retrogradation [36]. On the other hand, the fluctuating b* value indicates that Maillard-type browning [36] and structural rearrangements within the matrix likely occurred simultaneously. These color changes are consistent with the concurrent microbial and chemical deterioration observed, suggesting that visual attributes may serve as indirect indicators of quality loss during the post-cooking holding period. The partial masking of retrogradation-induced paling may have contributed to the stable L* values, resulting in apparent visual surface stability. However, the decline in YI indicates that pigment degradation and spoilage-related changes continued to progress within the product matrix. These findings suggest that color evolution in cooked bulgur is associated with starch retrogradation, pigment degradation, and temperature-dependent nonenzymatic reactions, all of which may contribute to the progressive deterioration of product quality during storage.

### 3.6. Changes in TBARS in Cooked Wheat

Lipid oxidation is a primary deterioration mechanism in food systems, leading to nutritional losses, mass reduction, and undesirable alterations in color, odor, and flavor that contribute to economic losses. Furthermore, lipid oxidation is widely recognized as a critical quality indicator because of its potential impact on consumer health [40]. Although wheat is not classified as a high-fat food matrix, the presence of volatile compounds such as hexanal and pentanal, along with increasing TBARS values, indicates that unsaturated fatty acids undergo oxidative degradation during bulgur processing and storage. Polyunsaturated fatty acids, such as linoleic acid, may be degraded via enzymatic pathways (e.g., lipoxygenase activity) or autoxidation, generating aldehydes such as hexanal and pentanal. These aldehydes are key chemical markers of lipid oxidation in cereal-based products [8,41].

TBARS values were determined after the post-cooking holding period to evaluate the effects of different holding temperatures on secondary lipid oxidation within the bulgur matrix. Table 6 summarizes the results.

Lipid oxidation in cereal-based systems is typically assessed using TBARS values, which measure MDA, a key secondary oxidation product and an indicator of cellular degradation and rancidity [42,43]. The kinetic behavior of TBARS values in the present study demonstrated a clear time-dependent intensification of lipid oxidation, in line with previous findings [32,44].

Elevated temperatures accelerate free radical formation and initiate the propagation phase of lipid autoxidation, which promotes the rapid conversion of matrix lipids into secondary volatile compounds, such as aldehydes, ketones, and MDA [38,43]. Furthermore, although water activity exerts a complex and specific effect on cereal matrices [45], the presence of moisture during post-cooking holding promotes oxidation by altering molecular mobility and the activity of pro-oxidant species.

The initial (t = 0) TBARS values of cooked wheat were 0.0573, 0.0521, and 0.0565 mg MDA/kg for samples held at 25, 35, and 45 °C, respectively. This result indicates that lipid oxidation initiated before the holding period, most likely as a result of tissue and cellular membrane disruption, which exposed lipids to oxygen [46].

Moreover, the analysis of TBARS kinetics revealed that the most notable increase occurred within the first 10 h of post-cooking holding, during which TBARS values approximately doubled. Subsequently, the oxidation rate declined between 10 and 50 h and approached a plateau. This trend indicates that lipid oxidation was most pronounced during the initial temperature-holding phase. Previous research has demonstrated that thermal processing disrupts cellular membrane integrity, exposing lipids to oxidative attack [47] and facilitating the rapid formation of primary and secondary oxidation products [36].

At the off-odor onset time, TBARS values showed only a modest decline with increasing holding temperature (0.1256, 0.1240, and 0.1205 mg MDA/kg at 25, 35, and 45 °C, respectively). By contrast, the relative peak area of lipid oxidation-derived volatile aldehydes (hexanal, nonanal, 2-nonenal, 2,4-heptadienal, and 2,4-decadienal) declined markedly at the same temperatures (5.78%, 4.33%, and 2.49%, respectively). This divergence, assessed on a time-matched basis, indicates that MDA and volatile aldehydes represent distinct downstream products of the same oxidative cascade, consistent with reports that MDA is a terminal lipid oxidation product in cereal grains. Aldehydes are generated as reactive intermediates during storage-associated lipid oxidation [42] and are subject to different physical and chemical fates as holding temperature increases. Different mechanisms may account for this temperature-dependent pattern. First, aldehydes are highly reactive electrophilic intermediates prone to further chemical transformation [48], and their apparent decline at higher holding temperatures may reflect progression toward more oxidized end products, not cessation of oxidative activity. This interpretation is consistent with the concurrent increase in hexadecanoic acid at 45 °C (from 0.43% at 25 °C to 3.03% at 45 °C, at the respective off-odor onset times; Table 7). Second, low-molecular-weight volatile aldehydes are generally more volatile than larger, less volatile oxidation products, such as MDA. Aroma compounds are more easily released into the headspace at higher processing temperatures, as the rise in temperature increases their activity coefficients and facilitates desorption [9]. By contrast, MDA is prone to covalent modification of matrix proteins, a mechanism documented in cereal-based systems undergoing lipid–protein co-oxidation during storage [38] and in model food protein systems [49]. This behavior may favor the retention of MDA within the grain matrix and its subsequent detection via the hot-acid hydrolysis step of the TBARS assay, despite ongoing turnover of the free aldehyde pool.

These results indicate that the reduced abundance of volatile aldehydes at elevated holding temperatures should be interpreted as a temperature-dependent shift in the oxidation product profile toward more advanced, matrix-associated, less volatile species. This shift, together with the concurrent decrease in YI and the simultaneous increase in b* values at 45 °C (discussed in Section 3.5), may indicate oxidative and nonenzymatic browning-related changes. Although no off-odor was detected at the start of the post-cooking holding (t = 0 h), the subsequent increase in TBARS values over the holding period suggests a concurrent deterioration process, in which both lipid oxidation and microbial activity likely contribute to the overall decline in bulgur quality within the matrix. Although TBARS provided a sensitive measure of secondary lipid oxidation across holding conditions, this assay was used alongside parallel GC–MS characterization of lipid-oxidation-derived volatile aldehydes (Table 7). Given the low lipid content of durum wheat, primary oxidation markers such as peroxide value and conjugated dienes were not prioritized. However, their inclusion in future research could clarify the earliest stages of oxidative onset and further complement the secondary-product profile characterized in the present work.

### 3.7. Changes in Volatile Aroma Compounds During Post-Cooking Holding

Cooking and the subsequent temperature-holding period are primary factors influencing the sensory acceptability of wheat samples. Table 7 presents the results of GC–MS analysis, which was conducted to evaluate the molecular basis of sensory quality and oxidative deterioration in wheat samples held at 25, 35, and 45 °C after cooking. The volatile profile comprises 74 compounds (aldehydes, alcohols, ketones, carboxylic acids, hydrocarbons, and furans), indicating extensive biochemical and thermal degradation during the post-cooking holding period. Degradation reactions include lipid oxidation, the Maillard reaction, and phenolic acid decarboxylation. These pathways are likely to interact synergistically [41].

Among the identified volatiles, 2-methoxy-4-vinylphenol (9.91–15.76%), dodecane (3.27–8.03%), tetradecane (6.30–7.40%), ethanol (1.05–12.66%), and acetoin (2.66–7.13%) exhibited the highest relative abundances and were the most dominant volatiles that defined the overall aroma and deterioration profile. The relative abundance of volatile compounds varied with temperature: 2-methoxy-4-vinylphenol exhibited the highest overall contribution, especially at 35 °C, whereas hydrocarbons, such as tetradecane and dodecane, were more abundant at 45 °C.

Aldehydes are mainly produced through lipid oxidation and subsequent degradation processes, but may also arise from Maillard-type reactions. These compounds contribute diverse sensory attributes, including green, grassy, sweet, citrus, fruity, and fatty notes. Because of their low odor thresholds, aldehydes are recognized as major contributors to the aroma of cereal-based products [41,50]. Most of these compounds originate from the oxidative cleavage of polyunsaturated fatty acids, such as linoleic and oleic acids, which are abundant in the wheat matrix [10,51].

Alcohols are derived from precursors such as polyunsaturated fatty acids and may react with organic acids to form esters that impart a fresh, floral aroma [52]. Heterocyclic compounds, including furans, pyrazines, thiophenes, thiazoles, pyrroles, imidazoles, and pyridines, are among the primary volatile aroma compounds generated by the Maillard reaction. These compounds contribute roasted, popcorn-like, and sweet flavor characteristics [50]. Alcohols are another notable class of compounds detected in cereals. These compounds may originate from the reduction of ketones or aldehydes produced during lipid peroxidation, or from microbial metabolism of proteins and amino acids [41]. The main alcohols detected in cooked wheat were ethanol, benzyl alcohol, and 2,3-butanediol (Table 7), with ethanol and benzyl alcohol being primarily associated with microbial metabolism [41]. Ketone compounds possess high odor thresholds and are produced via oxidation or thermal degradation of polyunsaturated fatty acids and by [46].

The GC–MS chromatograms revealed pronounced changes in compound concentrations during post-cooking holding. Two volatile compounds (2-methoxy-4-vinylphenol and hexanal) were consistently detected in all samples where odor was perceived. Both compounds were present at low concentrations at the onset of post-cooking holding (t = 0); however, their levels increased markedly during the holding period. Notably, by 50 h, 2-methoxy-4-vinylphenol increased markedly by 49.8-fold at 25 °C, 51.7-fold at 35 °C, and 46.5-fold at 45 °C. 2-Methoxy-4-vinylphenol arises from the decarboxylation of ferulic acid and is characterized by clove-like, smoky, and curry-like aroma notes. Ferulic acid is decarboxylated at elevated temperatures, likely explaining the occurrence of this compound in cooked cereals [9]. Similarly, 4-vinylphenol forms through the thermal decomposition of *p*-coumaric acid and has been identified as a contributor to undesirable odor in cereal matrices [53]. The detection of 4-vinylphenol in this study suggests that phenolic degradation may occur in the wheat matrix during cooking and post-cooking holding.

Zhao et al. [54] reported that several volatile compounds detected in cooked rice, including 1-octen-3-ol, 2-pentylfuran, (*E*)-2-heptenal, (*E*)-2-nonenal, 3-octen-2-one, hexanal, 2-octen-1-ol, 1-octanol, nonanal, 3,5-octadien-2-one, and octanal, originate from hydroperoxide decomposition. This finding indicates that soaking and cooking promote lipid oxidation, likely because of the increased activity of endogenous lipid-oxidizing enzymes, such as lipoxygenase and lipase [54]. Several of these oxidation-related volatiles, specifically 2-pentylfuran, 2-nonenal, 3,5-octadien-2-one, nonanal, and hexanal, were also identified in the present study. These results suggest that lipid oxidation progressed during the post-cooking holding period. Hexanal, 1-hexanol, and 3,5-octadien-2-one were identified as potential differential markers in raw wheat, resulting from lipid oxidation. However, only hexanal and 3,5-octadien-2-one exhibited marked variations in cooked wheat. Compared to its initial concentration, by 50 h, hexanal increased by 8.03-fold at 25 °C, 4.92-fold at 35 °C, and 5.06-fold at 45 °C. The increase in hexanal, a marker of lipid oxidation, during post-cooking holding indicates ongoing oxidative reactions within the system. This behavior further supports the TBARS-based evidence of lipid oxidation (see Section 3.6), indicating a consistency between chemical and volatile-based deterioration markers.

Among the identified volatile classes, aldehydes are considered the most typical secondary products of lipid oxidation [36] and play a crucial role in off-flavor development [4]. Hexanal and nonanal are primarily derived from the autoxidation of linoleic and oleic acids, respectively, and impart undesirable green, grassy, and fatty off-odors to the product [41,52]. Furthermore, they are likely responsible for the observed TBARS accumulation described in Section 3.6. Researchers have identified 3-(*E*)-2-decenal, (*E*,*E*)-2,4-nonadienal, (*E*,*E*)-2,4-decadienal, and (*E*)-2-nonenal as the primary aldehydes in rice [55]. Hexanal, octanal, nonanal, and decanal were identified in both raw oats and oat samples at various processing stages. (*E*,*E*)-2,4-decadienal was identified as one of the key compounds responsible for off-odor development [56]. Aldehydes also play a critical role in the aroma quality of processed cereal products. For instance, hexanal, octanal, pentanal, nonanal, heptanal, (*E*)-2-nonenal, (*E*,*E*)-2,4-nonadienal, and vanillin have been identified as key volatiles in cooked rice and rice porridge [41].

In addition to oxidative lipid degradation, the abundance of free carboxylic acids [36] suggests that hydrolytic rancidity associated with lipolytic activity may have progressed concurrently. Carboxylic acids, especially 2-pyridinepropanoic acid, tetradecanoic acid, and hexadecanoic acid, accumulated conspicuously, particularly at 45 °C. Previous studies on wheat volatiles have reported the presence of volatiles, including pentanoic, hexanoic, heptanoic, nonanoic, and octadecanoic acids [4]. In addition, Yousif et al. [22] reported that basic thermal operations involved in bulgur production, particularly cooking and drying, promote changes in the volatile profile and result in the formation of new compounds. The same study also identified decanoic acid in bulgur and associated it with rancid odor. Altogether, these findings suggest that elevated temperatures may promote hydrolytic and thermally induced deterioration in the bulgur matrix.

On the other hand, 2-pentylfuran was identified as one of the major volatiles associated with lipid oxidation that may contribute balsamic, cocoa-like, and coffee-like roasted notes to the product [8,46]. 2-Pentylfuran is thought to be formed through the cyclization of linoleic acid oxidation products [57].

Furthermore, the predominance of straight-chain hydrocarbons, particularly tetradecane and dodecane, implies that advanced thermal and oxidative degradation reactions occur within the wheat matrix. Although hydrocarbons, which have an unpleasant petrol-like odor, typically possess high odor thresholds and contribute minimally to aroma [32], their presence serves as a valuable chemical marker of extensive lipid deterioration.

Ketones, including 2,3-butanedione, acetoin were also abundant, and their total content increased with holding temperature. Ketones generally exhibit higher odor thresholds than aldehydes and are formed through multiple complex pathways, including the thermal oxidation of lipids, Maillard reactions, and Strecker degradation of amino acids [41,58]. Ketones are commonly linked to pleasant, sweet, buttery, and caramel-like aroma notes [59]. The substantial increase in ketone content observed during post-cooking holding at 45 °C indicates thermal degradation of the matrix and serves as a molecular marker of advanced Maillard reactions [60]. Ketone accumulation is linked to browning, as evidenced by the increase in the b* value, suggesting that elevated holding temperatures play a critical role in driving both oxidative rancidity and nonenzymatic flavor development in cooked bulgur.

Wheat was cooked at approximately 95–97 °C; thus, the grain’s initial microbial load was likely substantially reduced. However, the post-cooking moisture content (approximately 40–50%) provided a favorable environment for microbial growth. Nonetheless, the use of sterile containers to hold the samples and the initially low microbial population suggest that microorganisms require a certain lag phase before reaching sufficient numbers to provoke spoilage. This may explain why, in contrast to the shorter onset of odor formation observed after tempering, noticeable odor development in cooked samples occurred only after approximately 50 h. Therefore, the delayed onset of odor formation in cooked wheat samples appears to be associated not only with microbial activity, but also with time-dependent lipid oxidation and thermally induced reactions. Nevertheless, spoilage processes are likely to occur more rapidly under industrial conditions, where products are stored in bulk and exposed to environmental contamination.

Esters, such as ethyl acetate and isopropyl tetradecanoate, were also detected. Esters are formed through esterification reactions between acids and alcohols and are associated with floral and fruity aroma notes [41,46]. Because of their low odor thresholds, esters enhance the overall aroma profile of cereal-based products [46]. However, high holding temperatures, such as 35 or 45 °C, accelerate the oxidative degradation of polyunsaturated lipids, resulting in the accumulation of secondary oxidation products, such as aldehydes (e.g., hexanal, nonanal, and octanal) and furans (e.g., 2-pentylfuran). Aldehydes contribute fatty, green, and rancid notes, while furans impart heavier bean-like and burnt aroma attributes. Additionally, thermal processing can promote the decarboxylation of phenolic acids, particularly ferulic acid, promoting the formation of 2-methoxy-4-vinylphenol [9]. As a result, the mild fruity notes are likely to be masked by intense off-flavor compounds [8]. This outcome reflects the complex and synergistic interactions between oxidative and thermal degradation pathways within the bulgur matrix.

There is little information about the synergistic formation and combined effects of these lipid- and phenolic-derived volatile compounds during the cooking and post-cooking holding stages of bulgur production. Aroma formation and deterioration during cereal processing result from complex, interrelated pathways, such as lipid degradation, amino acid breakdown, and Maillard reactions. These processes may occur competitively or mutually promote each other [41].

The increasing diversity of volatile compounds suggests that the aroma profile experienced a progressive enrichment and growing complexity during the post-cooking holding period. However, at later stages, particularly at 45 °C, the number of detectable compounds declined, which may be attributed to volatilization losses and/or the secondary degradation or transformation of certain volatile constituents. The reduced abundance of hexanal and nonanal at 45 °C may be associated with the progression of lipid oxidation beyond the initial stages, where primary aldehydes are further transformed into secondary products, lost through volatilization, or involved in secondary reactions, such as Schiff base formation and Maillard-type interactions within the matrix [38].

A temperature of 35 °C appeared to be optimal for the simultaneous progression of enzymatic lipid oxidation and mesophilic microbial spoilage, resulting in the accumulation of volatile deterioration products, such as acetoin, 2-methoxy-4-vinylphenol, and 2-pentylfuran. Compounds such as 2-pentylfuran are widely associated with lipid oxidation and may also be influenced by concurrent thermally induced reaction pathways. On the other hand, 2-methoxy-4-vinylphenol is mainly produced by the decarboxylation of ferulic acid [9]. Notably, the relative abundance of these low-molecular-weight volatiles decreased when the incubation temperature increased to 45 °C, likely the outcome of enhanced volatilization losses. In addition, under higher thermal stress, reactive aldehydes and ketones may have been further consumed through secondary reactions within the matrix, including advanced nonenzymatic browning pathways and interactions with proteins. Thus, the decline in free aldehydes and other lipid oxidation products at later stages or under more severe temperature conditions may be associated with volatilization, oxidative polymerization, Schiff base formation, and lipid–protein co-oxidation [38]. Therefore, the apparent plateau observed in the TBARS data may not necessarily indicate a reduction in oxidative activity, but a dynamic balance between the formation of reactive carbonyls and their subsequent transformation or binding to matrix components.

### 3.8. Heat Map Analysis of the Volatile Compounds

**Figure 4:**
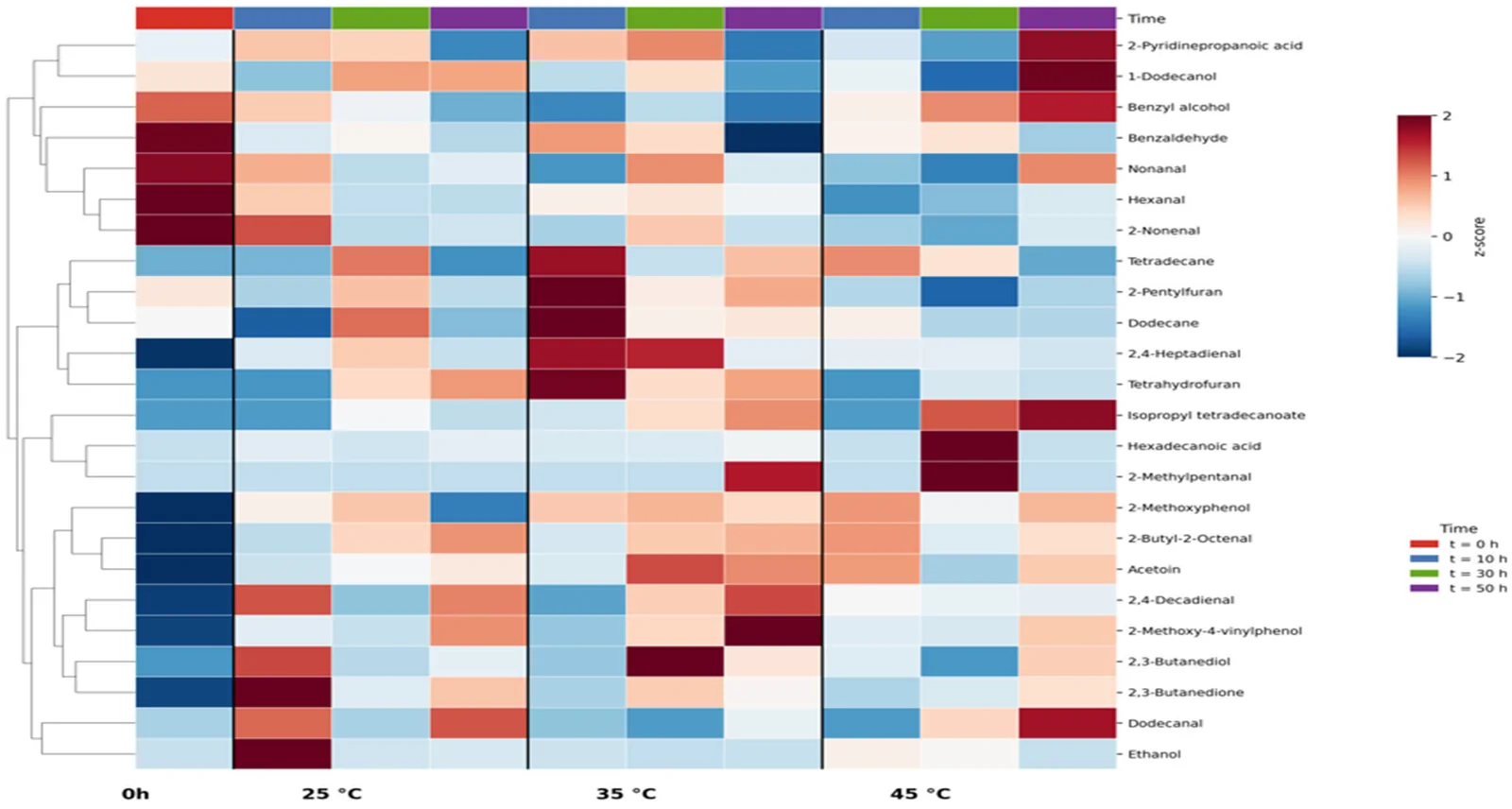
Heat map of volatile compounds detected in wheat during post-cooking holding at 25, 35, and 45 °C.

A heat map analysis was conducted to examine temporal and temperature-dependent changes in the volatile compound profiles of wheat during post-cooking holding at 25, 35, and 45 °C (Figure 4). Samples are organized horizontally by holding temperature and time, while volatile compounds are displayed vertically. The relative abundance of each compound is shown using a z-score-normalized color scale, with red representing concentrations above the overall mean and blue representing concentrations below the mean. Hierarchical clustering demonstrated that compounds segregated into three broad functional groups, each reflecting distinct deterioration pathways.

The first cluster, consisting of hexanal, nonanal, 2-nonenal, and benzaldehyde, showed the highest relative abundance at t = 0 h across all temperature groups, followed by a progressive decline during holding that was most pronounced at 45 °C. These compounds are established markers of lipid autoxidation in cereal-based systems, originating from both enzymatic and nonenzymatic degradation of unsaturated fatty acids [9]. Because these aldehydes are reactive electrophilic intermediates [48], their decline at elevated holding temperatures likely indicates a progression toward more oxidized, less volatile species. This interpretation aligns with the observed increase in hexadecanoic acid at 45 °C, consistent with the established role of carboxylic acids as terminal products of lipid oxidation in cereal matrices.

The second cluster, comprising acetoin, 2,3-butanediol, and 2,3-butanedione, together with ethanol, showed its most pronounced accumulation at 35 °C, particularly from 30 h onward. These compounds are characteristic products of the microbial butanediol fermentation pathway, in which pyruvate is converted via α-acetolactate to acetoin and subsequently to 2,3-butanediol, with spontaneous aerobic oxidation of acetoin yielding 2,3-butanedione [61]. This pathway is typically associated with facultatively anaerobic, spore-forming *Bacillus*-related genera, whose optimal growth range (up to 45–55 °C for thermotolerant strains) [61] is consistent with the pronounced microbial growth rate observed at 35 °C (Section 3.4). Therefore, the clustering of these compounds provides a volatile-level chemical signature that corroborates the temperature dependence of microbial activity inferred from the Gompertz modeling results. Notably, the aerobic oxidation step required for diacetyl formation is consistent with the residual interstitial oxygen within the sealed holding containers (Section 3.2), supporting a microaerophilic environment conducive to this fermentation pathway.

The third cluster, consisting of 2-methoxyphenol and 2-methoxy-4-vinylphenol, exhibited a progressive increase with both holding time and temperature, attaining the highest relative abundance under the most severe post-cooking holding conditions. 2-Methoxy-4-vinylphenol (4-vinylguaiacol) forms via the decarboxylation of ferulic acid, a phenolic acid abundant in wheat bran, through either thermal decomposition at elevated processing temperatures or microbial/enzymatic decarboxylation [62]. In our previous work on bulgur tempering, samples presenting off-odor were consistently characterized by elevated 2-methoxy-4-vinylphenol concentrations alongside the presence of *Aspergillus niger* [4]. Notably, the moisture content in the present study (~50%) considerably exceeds that of this earlier tempering work (23–27%), indicating an environment conducive to fungal proliferation. Members of the genus *Aspergillus* exhibit feruloyl esterase activity and are capable of releasing ferulic acid as a precursor of 2-methoxy-4-vinylphenol [63], suggesting that, alongside thermal decarboxylation, fungal metabolic activity may contribute to the accumulation of this compound under the elevated moisture and temperature conditions reported in the present study. The convergence of thermal and microbial pathways offers a plausible explanation for the consistent dominance of this compound across all three holding temperatures, a contribution that may be even more pronounced given the higher moisture content of the present system, as indicated by its high ROAVs (Section 3.9).

In conclusion, the heat map analysis demonstrates that the volatile profile of durum wheat during post-cooking holding evolves through at least three distinct deterioration pathways: lipid oxidation, microbial fermentation, and phenolic acid decarboxylation. The relative contributions of these pathways vary according to the holding temperature. This observation aligns with the broader interpretation presented in Section 3.4 and Section 3.7, Section 3.8 and Section 3.9, which suggests that deterioration at lower temperatures is primarily associated with microbial activity, whereas deterioration at the highest holding temperature may involve a greater relative contribution from chemical—including oxidative and thermal—pathways.

### 3.9. ROAVs of Key Volatile Compounds

ROAV analysis identified six compounds as major odor-active contributors (ROAV ≥ 1) across all three post-cooking holding temperatures: 2-methoxy-4-vinylphenol, 2,4-decadienal, 2-nonenal, 2,3-butanedione, 2-methoxyphenol, and nonanal (Table 8). Among these, 2-methoxy-4-vinylphenol consistently exhibited the highest ROAV. Notably, its relative peak area (8.62–15.76%) was not the highest among all detected volatiles. This disproportionate contribution can be attributed to its exceptionally low odor threshold (0.0017 mg/m^3^), indicating that even modest changes in its abundance may exert a pronounced influence on the perceived phenolic or smoky character of the samples. A similar trend was observed for 2,4-decadienal and 2-nonenal, both lipid oxidation-derived aldehydes with sub-microgram odor thresholds (0.0002 and 0.0005 mg/m^3^, respectively). Although their relative abundances remained below 1% throughout the holding period, their ROAVs (up to 33.5 and 35.5, respectively) suggest that these compounds may be associated with fatty, green, and rancid notes that may contribute meaningfully to off-odor perception. This effect is particularly notable during earlier holding stages at 25 °C, where both compounds exhibited their highest ROAVs.

By contrast, several compounds with comparatively high relative abundance appeared to have limited odor impact because of their considerably higher odor thresholds. Tetradecane and dodecane, which together accounted for more than 10% of the total peak area at all temperatures, yielded ROAVs below 0.03, suggesting a negligible direct contribution to the perceived aroma despite their quantitative dominance. Similarly, ethanol reached a relative abundance of 12.66% at 25 °C but exhibited a ROAV of only 0.003, consistent with its comparatively high odor threshold (93 mg/m^3^). Therefore, ethanol, while abundant, is unlikely to be a major driver of the alcohol-like notes that are sometimes associated with fermentative processes in cereal matrices. Acetoin followed a comparable pattern (ROAV ≤ 0.03 at all temperatures).

Among the modifier-class compounds (0.1 ≤ ROAV < 1), hexanal displayed a temperature-dependent decrease in ROAV from 1.157 at 25 °C (slightly above the key-compound threshold) to 0.217–0.252 at 35–45 °C. This trend may be associated with the progressive depletion or further oxidative transformation of hexanal into secondary lipid oxidation products, although the present data do not allow a definitive mechanistic conclusion. 2,4-Heptadienal and dodecanal, both potentially linked to fatty or green aroma notes, remained within the modifier range across temperatures. By contrast, 2-pentylfuran, often associated with green, earthy, or slightly musty notes in cereal-based products, showed a modest, relatively stable ROAV (0.195–0.300) that appeared largely independent of holding temperature. Octadecane appeared in the modifier range only at 45 °C, coinciding with its detection at this temperature. Given that long-chain alkanes are generally regarded as weak odorants, their sensory relevance remains uncertain.

Altogether, these findings suggest that the perceived off-odor of cooked durum wheat during post-cooking holding is primarily associated with a small subset of low-threshold compounds, specifically 2-methoxy-4-vinylphenol, 2,4-decadienal, and 2-nonenal. Despite their higher relative concentrations, compounds such as ethanol, acetoin, and the alkanes (decane, dodecane, tridecane, and tetradecane) appear to play a minimal sensory role, based on their calculated ROAVs. The results indicate a potential relationship between the qualitative changes in aroma detected during post-cooking holding and the temperature-dependent variations in a few odor-active markers. However, confirmation of the specific sensory attributes associated with each compound would require additional GC–olfactometry analyses.

The chosen controlled, sealed-container design isolates the intrinsic effect of holding conditions on deterioration kinetics. However, in open or bulk industrial environments, additional sources of microbial contamination, such as airborne microorganisms, equipment surfaces, and cross-contamination between batches, would likely result in higher initial microbial loads and a shorter lag phase than those observed in this study. Continuous oxygen availability in open systems may also accelerate lipid oxidation compared to the limited-headspace conditions applied here. Oxygen limitation at the core of a large bulk mass, combined with self-heating from microbial and enzymatic activity, could generate a more heterogeneous deterioration pattern than the homogeneous conditions modeled in this research. Therefore, future research incorporating pilot-scale, open, or forced-air storage trials is needed to clarify how our findings translate to industrial-scale practice.

## 4. Conclusions

This study found that quality deterioration in durum wheat during post-cooking holding at 25, 35, and 45 °C reflects a temperature-dependent interplay between microbial and chemical deterioration pathways. Bulk moisture content remained stable throughout holding, indicating that deterioration occurred under a sustained high-moisture condition rather than being driven by changes in moisture. Although microbial growth was fastest at 35 °C, off-odor onset occurred earliest at 45 °C, consistent with a shift toward more advanced lipid oxidation products and a sustained contribution of low-threshold phenolic and lipid-derived odorants, namely 2-methoxy-4-vinylphenol, 2,4-decadienal, 2-nonenal, 2,3-butanedione, and 2-methoxyphenol, despite their low relative abundance. These results indicate that microbial growth kinetics alone are insufficient to predict sensory shelf life in this system, and highlight the importance of evaluating chemical, microbiological, and odor-activity data at the actual point of sensory deterioration. This approach contributes to a limited body of evidence directly comparing the relative timing of microbial and chemical deterioration pathways within a single post-cooking holding system. From a practical standpoint, minimizing the duration of post-cooking holding at elevated temperatures, particularly those approaching 45 °C, may help delay off-odor onset even when microbial growth is limited. Nonetheless, these findings should be interpreted as reference values for open, bulk industrial storage. Future studies should evaluate the identified volatile markers as potential early indicators of impending off-odor onset under pilot-scale conditions and incorporate species-level microbial identification to further clarify the microbial contribution to the observed deterioration patterns.

## Figures and Tables

**Table 1:** Empirical models used to describe temporal changes in total moisture content during post-cooking holding.

Model	Equation	
Sigmoid	$y=y_{0}+\frac{a}{1+e^{-\left(\frac{X-X_{0}}{b}\right)}}\;$	(3)
Hill	y$\;=y_{0}+\frac{aX^{b}}{c^{b}+X^{b}}\;\;\;$	(4)

**Table 2:** Effect of cooking on the physical and chemical properties of wheat.

Property	*Triticum durum* (Raw)	*Triticum durum* (Cooked)
Moisture content (%, w.b.)	6.85 ± 0.15	50.17 ± 0.81
Ash content (%, d.b.)	1.55 ± 0.05	1.51 ± 0.43
Protein content (%, d.b.)	14.42 ± 0.21	13.12 ± 0.21
pH	6.98 ± 0.02	7.10 ± 0.02
L*	50.47 ± 0.22	49.90 ± 0.35
a*	7.97 ± 0.11	7.09 ± 0.11
b*	27.58 ± 0.41	30.52 ± 0.09
YI	78.08 ± 0.64	83.57 ± 0.54
TAMB-A (log CFU/g)	5.27 ± 0.10	1.36 ± 0.07

Values are expressed as the mean ± SD (*n* = 3); w.b.: wet basis; d.b.: dry basis; L*, a*, b*: CIELAB color parameters; YI: yellowness index; CFU: colony-forming unit.

**Table 3:** Parameters of the exponential model fitted to the titratable acidity data of wheat samples held at 25, 35, and 45 °C after cooking.

T (°C)	Y_0_	a	b	R^2^	RMSE
25 °C	1.488	0.844	0.298	0.958	0.012
35 °C	1.481	1.139	0.343	0.949	0.029
45 °C	1.479	1.610	0.739	0.983	0.009

**Table 4:** Maximum specific growth rate (μ_max_, h^−1^), lag phase (λ, h), Gompertz model parameters. a and b, and coefficient of determination (R^2^).

T (°C)	R^2^	RMSE	a	b	μ_max_	λ
25	0.997	0.0619	5.173	15.46	0.0339	0.245
35	0.999	0.0151	5.163	15.36	0.0651	0.242
45	0.993	0.0146	5.122	15.04	0.0352	0.234

**Table 5:** Color parameters (L*, a*, b*, and YI) of the wheat samples held at 25, 35, and 45 °C after cooking.

	L*			a*		
	**25 °C**	**35 °C**	**45 °C**	**25 °C**	**35 °C**	**45 °C**
0	46.62 ± 0.17 ^e^	48.17 ± 0.18 ^a,b,c^	47.38 ± 0.16 ^d^	7.95 ± 0.05 ^a^	7.76 ± 0.01 ^a,b,c,d^	7.93 ± 0.01 ^a^
10	47.67 ± 0.08 ^b,c,d^	48.23 ± 0.19 ^a,b,c^	47.30 ± 0.20 ^d^	7.89 ± 0.03 ^a,b^	7.84 ± 0.02 ^a,b^	7.82 ± 0.03 ^a,b,c^
20	48.36 ± 0.06 ^a,b^	48.23 ± 0.16 ^a,b,c^	47.59 ± 0.13 ^c,d^	7.82 ± 0.02 ^a,b,c^	7.73 ± 0.03 ^a,b,c,d^	7.71 ± 0.02 ^a,b,c,d^
30	48.35 ± 0.12 ^a,b^	48.29 ± 0.08 ^a,b,c^	47.64 ± 0.13 ^c,d^	7.63 ± 0.03 ^b,c,d^	7.82 ± 0.03 ^a,b,c^	7.87 ± 0.03 ^a,b^
50	48.55 ± 0.16 ^a^	48.60 ± 0.04 ^a^	47.79 ± 0.11 ^b,c,d^	7.49 ± 0.01 ^d^	7.55 ± 0.02 ^c,d^	7.78 ± 0.04 ^a,b,c^
	b*			**YI**		
0	32.30 ± 0.02 ^b,c,d,e^	32.10 ± 0.16 ^c,d,e^	30.30 ± 0.01 ^g^	89.60 ± 0.04 ^a^	88.86 ± 0.03 ^a,b^	89.53 ± 0.06 ^a^
10	31.93 ± 0.03 ^e^	32.78 ± 0.05 ^a,b^	31.29 ± 0.04 ^f^	89.85 ± 0.03 ^a^	88.86 ± 0.01 ^a,b^	88.70 ± 0.01 ^a,b^
20	32.57 ± 0.00 ^a,b,c,d^	33.10 ± 0.04 ^a^	30.60 ± 0.03 ^g^	88.80 ± 0.02 ^a,b^	87.78 ± 0.28 ^b,c^	87.59 ± 0.02 ^b,c^
30	32.90 ± 0.02 ^a^	32.00 ± 0.01 ^d,e^	32.60 ± 0.05 ^a,b,c^	87.72 ± 0.01 ^b,c^	86.38 ± 0.02 ^c,d^	86.54 ± 0.05 ^c,d^
50	32.30 ± 0.01 ^b,c,d,e^	32.02 ± 0.01 ^d,e^	32.70 ± 0.03 ^a,b^	87.48 ± 0.03 ^b,c^	85.93 ± 0.26 ^d^	85.67 ± 0.01 ^d^

Values are expressed as mean ± standard deviation. Different letters within the same row indicate significant differences (*p* < 0.05).

**Table 6:** TBARS values (mg MDA/kg) of wheat samples held at 25, 35, and 45 °C after cooking.

TBARS (mg MDA/kg)			
Time (h)	25 °C	35 °C	45 °C
0	0.0573 ± 0.006 ^a^	0.0521 ± 0.004 ^a^	0.0565 ± 0.005 ^a^
10	0.1079 ± 0.001 ^b^	0.1092 ± 0.002 ^bc^	0.1068 ± 0.006 ^b^
20	0.1087 ± 0.003 ^bc^	0.1149 ± 0.008 ^bcd^	0.1133 ± 0.002 ^bc^
50	0.1256 ± 0.000 ^d^	0.1258 ± 0.003 ^d^	0.1292 ± 0.004 ^de^

Values are expressed as mean ± standard deviation. Different letters within the same row indicate. significant differences (*p* < 0.05).

**Table 7:** Volatile aroma compounds identified by GC–MS in wheat samples with detectable off-odor after post-cooking holding at 25, 35 and 45 °C.

RT (min)	Compounds	Odor Description *	MW (g/mol)	Relative Abundance 25 °C (%)	Relative Abundance 35 °C (%)	Relative Abundance 45 °C (%)
Carboxylic acids						
1.147	2-Pyridinepropanoic acid	Acidic, rancid	151.16	3.25	4.00	3.50
69.411	Tetradecanoic acid	Waxy, fatty	228.37	n.d.	n.d.	0.46
73.519	Pentadecanoic acid	Waxy	242.4	n.d.	n.d.	0.26
77.295	Hexadecanoic acid	Waxy	256.42	0.43	n.d.	3.03
83.921	Octadecanoic acid	Mild fatty, waxy	284.5	n.d.	n.d.	0.29
Aldehydes						
1.450	2-Methylpentanal	Ethereal	100.16	n.d.	n.d.	1.26
5.404	Hexanal	Green, grass	100.16	1.76	0.70	0.38
13.481	Benzaldehyde	Almond	106.12	1.00	0.85	1.70
17.078	2,4-Heptadienal	Green, fatty	110.15	0.55	0.74	0.86
23.925	Nonanal	Citrus, green	142.24	2.23	1.99	0.64
27.861	2-Nonenal	Green	140.22	0.90	0.54	0.28
40.435	2,4-Decadienal	Fried fat	152.23	0.34	0.36	0.33
44.820	2-Butyl-2-octenal	Green, fatty	182.30	1.15	1.40	1.06
47.391	Dodecanal	Waxy, citrus	184.32	0.41	n.d.	0.32
67.134	Pentadecanal	Fresh waxy	226.4	n.d.	n.d.	0.25
Alcohols						
1.314	Ethanol	Vinous, pungent	46.07	12.66	1.05	4.44
2.870	1,2-Ethanediol	Odorless	62.07	n.d.	n.d.	0.37
18.618	2-Ethyl-1-hexanol	Citrus	130.23	n.d.	0.31	0.25
18.723	Benzyl alcohol	Floral	108.14	0.28	0.21	0.75
4.820	2,3-Butanediol	Fruity	90.12	0.35	0.21	n.d
30.404	1-Dodecanol	Wax, sweet	186.33	0.76	1.68	n.d.
46.301	1-Hexadecanol	Waxy, greasy	242.44	n.d.	0.46	n.d.
Phenols						
22.624	2-Methoxyphenol	Phenolic	124.14	0.61	1.23	0.77
40.116	2-Methoxy-4-vinylphenol	Spicy, smoky	150	8.62	15.76	9.91
Esters						
1.926	Ethyl acetate	Ethereal	88.1	0.60	0.34	1.1
17.580	Hexyl acetate	Fruity, green	144.21	n.d.	0.21	0.59
37.125	2-Methylpropyl heptanoate	Herbal, fruity	186.29	n.d.	0.23	n.d.
38.560	2-Ethylhexyl hexanoate	Odorless	228.37	n.d.	0.91	0.42
72.155	Isopropyl tetradecanoate	Oily, fatty	270.5	n.d.	n.d.	0.26
73.189	Diethyl octanedioate	Stewed apple	230.30	n.d.	n.d.	0.28
Furans						
2.055	Tetrahydrofuran	Ethereal, musty	72.11	n.d.	n.d.	0.28
15.846	2-Pentylfuran	Green bean	138	2.13	2.68	1.59
Hydrocarbons						
6.167	3,4,5-Trimethylheptane	Faint, petroleum	142.28	n.d.	0.36	n.d.
9.375	Styrene	Balsamic	104.15	0.23	0.22	n.d.
16.489	Decane	Gasoline	142.28	0.60	0.62	n.d.
17.479	2,5-Dimethylnonane	Sweet, nutty	156.31	0.18	0.66	0.51
18.079	4,6-Dimethyldodecane	Gasoline, oily	198.39	2.01	0.77	0.58
20.589	5-Methylundecane	Waxy, sweet	170.33	n.d.	n.d.	1.88
20.972	5-(2-Methylpropyl)nonane	Faint, fatty	170.33	0.30	0.39	0.34
30.848	1-Dodecene	Olefinic	168.32	n.d.	n.d.	0.42
31.089	Dodecane	Oily petroleum	170.33	6.08	8.03	3.27
31.486	2-Methyltetracosane	Odorless	352.7	n.d.	1.36	0.46
32.486	2,5-Dimethylundecane	Skunky, yeasty	184.36	n.d.	0.46	0.47
33.000	4-Methyldodecane	Waxy, oily	184.36	0.33	0.78	0.31
34.895	Eicosane	Odorless	310.6	1.17	1.10	1.67
35.817	4,6-Dimethyldodecane	Waxy, oily	198.39	0.48	1.41	1.24
36.550	5-Methyltetradecane	Mild, waxy	212.41	n.d.	n.d.	0.73
37.872	8-Methylheptadecane	Petroleum-like	254.5	2.61	2.38	1.88
39.465	Tridecane	Gasoline	184.36	1.23	1.58	0.59
39.560	Tricosane	Waxy, fatty	324.6	n.d.	n.d.	0.22
46.880	Tetradecane	Mild waxy	198.39	6.72	7.40	6.30
51.134	2,6,10,14-Tetramethylhexadecane	Odorless	282.55	0.30	0.71	0.68
53.012	Docosane	Odorless	310.6	0.26	0.51	0.64
53.730	Heneicosane	Waxy	296.60	1.53	1.21	1.79
56.936	Hentriacontane	Odorless	436.8	n.d.	n.d.	0.28
65.514	Heptadecane	Odorless	240.5	1.05	n.d.	0.47
71.041	Hexacosane	Waxy, fatty	366.7	n.d.	n.d.	0.39
75.031	Octadecane	Faint, fuel-like	254.5	n.d.	n.d.	0.26
Ketones						
1.806	2,3-Butanedione	Butter, popcorn	86.09	1.83	2.51	1.52
3.042	Acetoin	Buttery	88.11	3.90	7.13	2.66
23.075	3,5-Octadien-2-one	Fatty	124.18	n.d.	n.d.	0.34
50.377	6,10-Dimethyl-5,9-undecadien-2-one	Floral, fruity	194.31	n.d.	n.d.	0.35
52.437	6-Acetyltetralin	Floral	174.24	0.79	1.19	2.01
66.403	2-Pentadecanone	Spicy, green, hay-like	226.4	n.d.	n.d.	0.34
72.853	6,10,14-Trimethyl-2-pentadecanone	Floral, woody	268.48	0.83	0.95	1.48
Lactones						
81.990	5-Dodecyldihydro-2(3H)-furanone	Sweet, creamy	254.41	n.d.	n.d.	0.81
83.025	Delta-tridecalactone	Creamy	212.33	n.d.	n.d.	0.36
Terpenes						
18.228	D-Limonene	Citrus	136.23	n.d.	n.d.	0.42

* Odor classifications were obtained from PubChem.

**Table 8:** Relative odor activity values (ROAVs) of key volatile compounds in durum wheat during post-cooking holding at 25, 35, and 45 °C.

Compounds	C% 25 °C	C% 35 °C	C% 45 °C	ROAV 25 °C	ROAV 35 °C	ROAV 45 °C	Threshold (mg/m^3^)
2-Methoxy-4-vinylphenol	8.62	15.76	9.91	100.000	100.000	100.000	0.0017
2,4-Decadienal	0.34	0.36	0.33	33.527	19.416	28.305	0.0002
2-Nonenal	0.90	0.54	0.28	35.499	11.650	9.606	0.0005
2,3-Butanedione	1.83	2.51	1.52	9.023	6.769	6.519	0.004
2-Methoxyphenol	0.61	1.23	0.77	4.010	4.423	4.403	0.003
Nonanal	2.23	1.99	0.64	4.398	2.147	1.098	0.01
2,4-Heptadienal	0.55	0.74	0.86	0.190	0.140	0.259	0.057
Octadecane	0.00	0.00	0.26	0.000	0.000	0.223	0.02
Hexanal	1.76	0.70	0.38	1.157	0.252	0.217	0.03
2-Pentylfuran	2.13	2.68	1.59	0.300	0.206	0.195	0.14
Dodecanal	0.41	0.00	0.32	0.245	0.000	0.166	0.033
Benzaldehyde	1.00	0.85	1.70	0.039	0.018	0.058	0.5
2-Methylpentanal	0.00	0.00	1.26	0.000	0.000	0.054	0.4
D-Limonene	0.00	0.00	0.42	0.000	0.000	0.053	0.135
Tetradecane	6.72	7.40	6.30	0.027	0.016	0.022	5
Acetoin	3.90	7.13	2.66	0.027	0.027	0.016	2.9
Hexyl acetate	0.00	0.21	0.59	0.000	0.003	0.012	0.86
Ethyl acetate	0.60	0.34	1.10	0.003	0.001	0.005	3.6
Dodecane	6.08	8.03	3.27	0.010	0.007	0.005	11.8
Ethanol	12.66	1.05	4.44	0.003	0.000	0.001	93
Tridecane	1.23	1.58	0.59	0.001	0.000	0.000	42
Styrene	0.23	0.22	0.00	0.032	0.017	0.000	0.14
Decane	0.60	0.62	0.00	0.002	0.001	0.000	7.45

## Data Availability

The original contributions presented in this study are included in the article/Supplementary Materials. Further inquiries can be directed to the corresponding author.

## Supplementary Materials

The following supporting information can be downloaded at: https://www.mdpi.com/article/10.3390/foods15172983/s1, Figure S1: (a) Sigmoid and (b) Hill models describing moisture changes in cooked wheat during post-cooking incubation at 25, 35, and 45 °C. Table S1: Parameters of the sigmoid and Hill models fitted to moisture contents of wheat samples held at 25, 35, and 45 °C after cooking.

## References

[1] Bay Yılmaz B., Bayram M., Türker N. (2025). Modeling water absorption of intact bulgur during tempering at different temperatures and moisture levels. J. Food Sci..

[2] Michel S., Bayram M. (2024). Influence of cooking water hardness on the chemical, colour and textural characteristics of bulgur at different processing stages. J. Cereal Sci..

[3] Stone A.K., Wang S., Tulbek M., Koksel F., Nickerson M.T. (2020). Processing and quality aspects of bulgur from *Triticum durum*. Cereal Chem..

[4] Bay Yılmaz B., Bayram M., Türker N. (2025). Impact of tempering conditions on special spoilage, off-odor and the formation of volatile compounds in bulgur. Food Res. Int..

[5] Ma M., Sun Q.J., Li M., Zhu K.X. (2020). Deterioration mechanisms of high-moisture wheat-based food—A review from physicochemical, structural, and molecular perspectives. Food Chem..

[6] Zhang Q., Xia S., Li J., Zhang X., Yu J. (2020). Effect of moisture transfer on texture uniformity of cooked rice after heat preservation with electric rice cooker. J. Cereal Sci..

[7] Qiao C.C., Tian X.H., Wang L.X., Jiang P., Zhai X.T., Wu N.N., Tan B. (2022). Quality characteristics, texture properties and moisture migration of fresh brown rice noodles under different storage and temperatures conditions. J. Cereal Sci..

[8] Hu X., Fang C., Zhang W., Lu L., Guo Z., Li S., Chen M. (2023). Change in volatiles, soluble sugars and fatty acids of glutinous rice, japonica rice and indica rice during storage. LWT.

[9] Lina G., Min Z. (2022). Formation and release of cooked rice aroma. J. Cereal Sci..

[10] Jin W., Zhao S., Sun H., Pei J., Gao R., Jiang P. (2023). Characterization and discrimination of flavor volatiles of different colored wheat grains after cooking based on GC-IMS and chemometrics. Curr. Res. Food Sci..

[11] Tian X., Wu F., Zhou G., Guo J., Liu X., Zhang T. (2023). Potential volatile markers of brown rice infested by the rice weevil, *Sitophilus oryzae* L. Coleoptera: Curculionidae. Food Chem. X.

[12] Sabri N.S.M., Mahyudin N.A., Sani M.S.A., Han M.G., Chong K.H., Padmanabhan K., Shan J., Rashid N.K.M.A. (2024). Microbial populations, sensory, and volatile compounds profiling of local cooked rice. Food Qual. Saf..

[13] Bayram M. (2005). Modelling of cooking of wheat to produce bulgur. J. Food Eng..

[14] Dayıoğlu İ. (2019). Solid Handling Properties of Bulgur Produced by Antep and Karaman/Mut Techniques. Master’s Thesis.

[15] (2012). Cereals—Determination of Bulk Density, Called Mass Per Hectolitre—Part 3: Routine Method.

[16] (1999). Approved Methods of Analysis.

[17] (1999). Approved Methods of Analysis.

[18] (1999). Approved Methods of Analysis.

[19] Elgün A., Ertugay Z. (1995). Tahıl İşleme Teknolojisi.

[20] Bozkurt H. (2007). Comparison of the effects of sesame and *Thymbra spicata* oil during the manufacturing of Turkish dry-fermented sausage. Food Control.

[21] Gao C., Li Y., Pan Q., Fan M., Wang L., Qian H. (2021). Analysis of the key aroma volatile compounds in rice bran during storage and processing via HS-SPME GC/MS. J. Cereal Sci..

[22] Yousif S.I., Bayram M., Kesen S. (2018). Characterization of volatile compounds of bulgur Antep type produced from durum wheat. J. Food Qual..

[23] van Gemert L.J. (2011). Odour Thresholds: Compilations of Odour Threshold Values in Air, Water and Other Media.

[24] Wang L., Zhang R., Wu H., Xie J., Tang Q., Dong Z. (2025). Sensory–Chemical Co-Dynamics in *Kadsura coccinea*: ROAV-Driven Prioritization of Cultivar-Specific Odorants and Mechanistic Validation via Molecular Docking. Foods.

[25] Horigane A.K., Naito S., Kurimoto M., Irie K., Yamada M., Motoi H., Yoshida M. (2006). Moisture distribution and diffusion in cooked spaghetti studied by NMR imaging and diffusion model. Cereal Chem..

[26] Ozturk O.K., Takhar P.S. (2018). Water Transport in Starchy Foods: Experimental and Mathematical Aspects. Trends Food Sci. Technol..

[27] Peleg M. (2023). Empirical Models of Sigmoid and Non-Sigmoid Hydration and Moisture Sorption Curves. Food Eng. Rev..

[28] Yang W., Zhu K., Guo X. (2022). Effect of bacteria content in wheat flour on storage stability of fresh wet noodles. Foods.

[29] Li M., Ma M., Zhu K.X., Guo X.N., Zhou H.M. (2017). Critical conditions accelerating the deterioration of fresh noodles: A study on temperature, pH, water content, and water activity. J. Food Process. Preserv..

[30] Jia X., Luo X., Li Y., Ji Z., Zhang H., Shen W. (2026). Understanding the quality changes and underlying mechanism of wheat-stewed dough during storage. Ital. J. Food Sci..

[31] Ehling-Schulz M., Frenzel E., Gohar M. (2015). Food-bacteria interplay: Pathometabolism of emetic *Bacillus cereus*. Front. Microbiol..

[32] Qu L., Zhao Y., Li Y., Lv H. (2025). Effect of storage temperature on the quality of brown rice revealed by integrated GC–MS and lipidomics analysis. Food Chem..

[33] Rocha-Villarreal V., Serna-Saldivar S.O., García-Lara S. (2018). Effects of parboiling and other hydrothermal treatments on the physical, functional, and nutritional properties of rice and other cereals. Cereal Chem..

[34] Tamura M., Singh J., Kaur L., Ogawa Y. (2019). Effect of post-cooking storage on texture and in vitro starch digestion of Japonica rice. J. Food Process Eng..

[35] Liu M., Xu M., Chen L., Zhuang K., Chen X., Chang X., Ding W. (2025). Effects of moderate retrogradation on the physical-chemical properties, water distribution and micro-structure of a Chinese traditional rice/bean food Dousi. LWT.

[36] Kilic-Buyukkurt O., Guclu G., Keskin M., Kelebek H., Selli S. (2025). Comparative study of the aroma profiles and bioactive properties of cooked and uncooked regular and firik bulgurs. Cereal Chem..

[37] Yilmaz V.A. (2019). Effects of different cooking and drying methods on phenolic acids, carotenoids, and antioxidant activity of emmer (*Triticum turgidum* ssp. *dicoccum*) bulgur. Cereal Chem..

[38] Yu T., Li Y., Luo R., Gao S., Jiang Q., Ma M., Zhang L., Xiao Y. (2026). Analysis of quality deterioration of buckwheat hele noodles during freezing storage: Oxidation of lipids and proteins. Food Chem..

[39] Pekkirişci B., Bilgiçli N., Cankurtaran-Kömürcü T. (2023). Determination of the physical, chemical, and antinutritional properties of firik and einkorn bulgur. Cereal Chem..

[40] Kanatt S.R., Chawla S.P. (2018). Shelf life extension of chicken packed in active film developed with mango peel extract. J. Food Saf..

[41] Feng Y., Cao H., Song H., Huang K., Zhang Y., Zhang Y., Li S., Li Y., Lu J., Guan X. (2024). The formation mechanism, analysis strategies and regulation measures of cereal aroma: A review. Trends Food Sci. Technol..

[42] Liu K., Zhang C., Xu J., Liu Q. (2021). Research advance in gas detection of volatile organic compounds released in rice quality deterioration process. Compr. Rev. Food Sci. Food Saf..

[43] Wang H., Zhu S., Ramaswamy H.S., Hu F., Yu Y. (2018). Effect of high pressure processing on rancidity of brown rice during storage. LWT.

[44] Rashid M.T., Liu K., Han S., Jatoi M.A., Sarpong F. (2023). Optimization of extrusion treatments, quality assessments, and kinetics degradation of enzyme activities during storage of rice bran. Foods.

[45] Mora R.L., Vanare S.P., Pegg R.B. (2025). Mechanisms, causes, and solutions: A comprehensive review of lipid oxidation in low-moisture packaged snacks. Eur. J. Lipid Sci. Technol..

[46] Zhang W., Yang Y., Zhang J., Zheng W., Du Y., Dang B. (2024). Study of the effect of milling on nutritional and sensory quality and volatile flavor compounds of cooked highland barley rice. LWT.

[47] Chemat A., Song M., Li Y., Fabiano-Tixier A.S. (2023). Shade of innovative food processing techniques: Potential inducing factors of lipid oxidation. Molecules.

[48] Zhao M., Liu Z., Zhang W., Xia G., Li C., Rakariyatham K., Zhou D. (2025). Advance in aldehydes derived from lipid oxidation: A review of the formation mechanism, attributable food thermal processing technology, analytical method and toxicological effect. Food Res. Int..

[49] Wang J., Zhao M., Qiu C., Sun W. (2018). Effect of malondialdehyde modification on the binding of aroma compounds to soy protein isolates. Food Res. Int..

[50] Hu X., Lu L., Guo Z., Zhu Z. (2020). Volatile compounds, affecting factors and evaluation methods for rice aroma: A review. Trends Food Sci. Technol..

[51] Bassam S.M., Noleto-Dias C., Farag M.A. (2022). Dissecting grilled red and white meat flavor: Its characteristics, production mechanisms, influencing factors and chemical hazards. Food Chem..

[52] Qiang W., Sun H., Huang X., Song H., Ying X., Wang X., Li Y., Zhang R., Xue H. (2025). Analysis of aroma-active compounds in different wheat flour mill streams using dynamic headspace extraction and comprehensive two-dimensional gas chromatography–olfactometry–mass spectrometry. J. Food Compos. Anal..

[53] Zeng Z., Zhang H., Jei Y.C., Zhang T., Matsunaga R. (2007). Direct extraction of volatiles of rice during cooking using solid-phase microextraction. Cereal Chem..

[54] Zhao Q., Xi J., Xu D., Jin Y., Wu F., Tong Q., Yin Y., Xu X. (2022). A comparative HS-SPME/GC-MS-based metabolomics approach for discriminating selected japonica rice varieties from different regions of China in raw and cooked form. Food Chem..

[55] Verma D.K., Srivastav P.P. (2020). A paradigm of volatile aroma compounds in rice and their product with extraction and identification methods: A comprehensive review. Food Res. Int..

[56] McGorrin R.J. (2019). Key aroma compounds in oats and oat cereals. J. Agric. Food Chem..

[57] Zhou Y., Gao S., Wei J., Chen X., Zhu S., Zhou X. (2023). Systematical construction of rice flavor types based on HS-SPME-GC–MS and sensory evaluation. Food Chem..

[58] Wei C.K., Ni Z.J., Thakur K., Liao A.M., Huang J.H., Wei Z.J. (2020). Aromatic effects of immobilized enzymatic oxidation of chicken fat on flaxseed (*Linum usitatissimum* L.) derived Maillard reaction products. Food Chem..

[59] Starowicz M., Zieliński H. (2019). How Maillard reaction influences sensorial properties (color, flavor and texture) of food products?. Food Rev. Int..

[60] Cui H., Yu J., Zhai Y., Feng L., Chen P., Hayat K., Xu Y., Zhang X., Ho C.T. (2021). Formation and fate of Amadori rearrangement products in Maillard reaction. Trends Food Sci. Technol..

[61] Xiao Z., Wang X., Huang Y., Huo F., Zhu X., Xi L., Lu J.R. (2012). Thermophilic fermentation of acetoin and 2,3-butanediol by a novel *Geobacillus* strain. Biotechnol. Biofuels..

[62] Xu L., Zhang H., Cui Y., Zeng D., Bao X. (2020). Increasing the Level of 4-Vinylguaiacol in Top-Fermented Wheat Beer by Secretory Expression of Ferulic Acid Decarboxylase from *Bacillus pumilus* in Brewer’s Yeast. Biotechnol. Lett..

[63] Todokoro T., Negoro H., Kotaka A., Hata Y., Ishida H. (2022). *Aspergillus oryzae* FaeA Is Responsible for the Release of Ferulic Acid, a Precursor of Off-Odor 4-Vinylguaiacol, in Sake Brewing. J. Biosci. Bioeng..

