# Supplementary material for: Post-Cooking Quality Deterioration of Wheat (Triticum durum) During Bulgur Production
Source: Foods. 2026 Aug 25;15(17):2983. doi: 10.3390/foods15172983 (PMC13564736; doi:10.3390/foods15172983)
Supplement: Supplementary file 1 [file foods-15-02983-s001.zip › foods-4491907-supplementary.pdf]

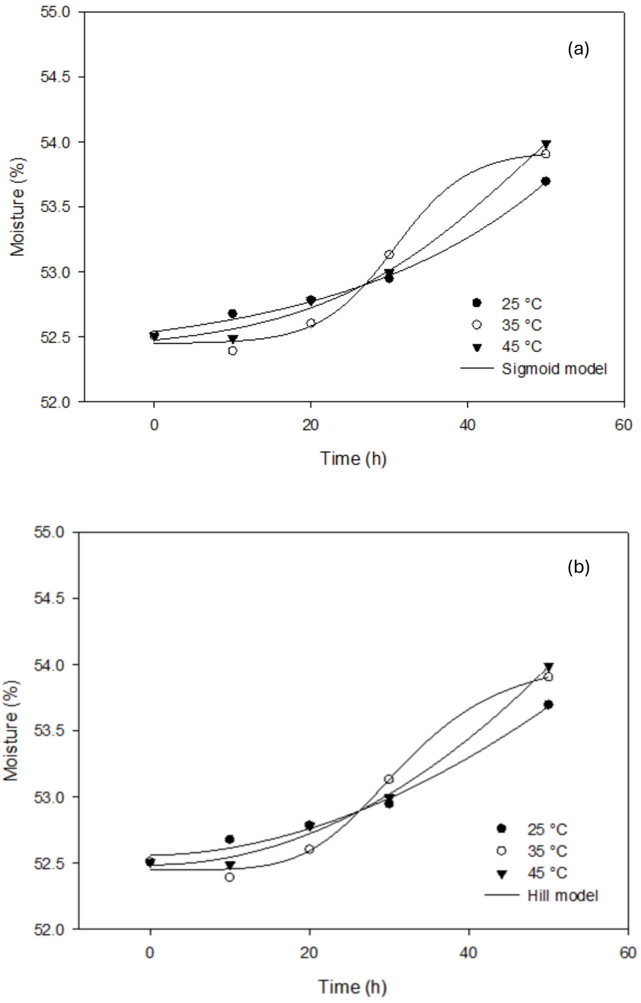

Figure S1. (a) Sigmoid and (b) Hill models describing moisture changes in cooked wheat during post-cooking incubation at 25, 35, and 45 °C.

Table S1. Parameters of the sigmoid and Hill models fitted to moisture contents of wheat samples held at 25, 35, and 45 °C after cooking.

Model	Parameter	Value	R^2	RMSE
	a	2.521e+0		
**Sigmoid model**	b	1.436e+1	0.992	0.006
(25 °C)	X_0	5.171e+1		
	y_0	5.251e+1		
	a	1.397e+0		
**Sigmoid model**	b	2.417e+0	0.986	0.025
(35 °C)	X_0	3.106e+1		
	y_0	5.251e+1		
	a	1.871e+0		
**Sigmoid model**	b	8.617e+0	0.991	0.014
(45 °C)	X_0	3.853e+1		
	y_0	5.250e+1		
	a	2.325e+0		

**Hill model**	b	2.424e+0	0.971	0.024
(25 ℃)	c	5.012e+1		
	y_0	5.251e+1		
	a	1.407e+0		
**Hill model**	b	1.198e+1	0.982	0.031
(35 ℃)	c	2.992e+1		
	y_0	5.246e+1		
	a	2.946e+0		
**Hill model**	b	2.936e+0	0.992	0.011
(45 ℃)	c	5.070e+1		
	y_0	5.249e+1		
